# Proteome-Wide Analysis and Surface Protein Isolation for Secretome Characterization Reveal Insights into the Biology of the Leaf-Cutter Ant *Acromyrmex echinatior*

**DOI:** 10.1021/acs.analchem.5c05220

**Published:** 2025-12-11

**Authors:** Penghsuan Huang, Joseph Sardina, Haiyan Lu, Gaspar Bruner-Montero, Cameron R. Currie, Lingjun Li

**Affiliations:** † Department of Chemistry, 5228 University of Wisconsin-Madison, Madison, Wisconsin 53706, United States; ‡ Department of Bacteriology, University of Wisconsin-Madison, Madison, Wisconsin 53706, United States; § School of Pharmacy, University of Wisconsin-Madison, Madison, Wisconsin 53705, United States; ∥ 3710 Estación Científica Coiba-AIP, Ciudad Del Saber, Clayton, Panamá 0816-02852, Panama; ⊥ Department of Biochemistry & Biomedical Sciences, Mcmaster University, Hamilton, Ontario L8S 4K1, Canada; # Centro de Biodiversidad Y Descubrimiento de Drogas, Instituto de Investigaciones Científicas Y Servicios de Alta Tecnología-AIP (INDICASAT-AIP), Ciudad Del Saber, Clayton, Panamá 0843-01103, Panama; ∇ Sistema Nacional de Investigación (SNI), Secretaría Nacional de Ciencia tecnología E Innovación (SENACYT), Ciudad Del Saber, Clayton, Panamá 0816-02852, Panama; ○ Lachman Institute for Pharmaceutical Development, School of Pharmacy, University of Wisconsin−Madison, Madison, Wisconsin 53705, United States; ◆ Wisconsin Center for nanobiosystems, School of Pharmacy, University of Wisconsin−Madison, Madison, Wisconsin 53705, United States

## Abstract

Characterizing the proteome of an organism can provide critical insights into the proteins that regulate key biological processes such as development, physiology, and environmental interactions. While proteome-wide analyses reveal broad protein dynamics, spatially resolved approaches can uncover specific, localized functions. For example, the leaf-cutter ant *Acromyrmex echinatior* secretes a unique protein layer that coats its exoskeleton and interacts with biotic and abiotic factors, including its symbiotic bacterium *Pseudonocardia*. In this study, to characterize both the whole-body proteome and the externally secreted cuticular protein layer of *A. echinatior*, we utilize a dual-layered proteomic approach. Using diaPASEF, we quantified 4,428 proteins across four early adult ages, uncovering distinct age-dependent protein clusters enriched in muscle development, lipid metabolism, and immune-related responses. We then developed an acid-based extraction method to isolate the externally secreted protein layer, identifying 323 secreted proteins via the ddaPASEF acquisition. Many of these proteins exhibited temporal abundance changes and were associated with functions, such as environmental stress response, microbial defense, and cuticle sclerotization. Notably, tropomyosin-family proteins were highly enriched in the external secretome and exhibited significant changes across early adult time points, potentially linking these ion-binding molecules to metal-enrichment processes occurring during this crucial stage. Together, this work reveals dynamic changes in the internal and surface proteomes of young adult *A. echinatior* ants and provides a methodological framework for further probing localized extra-cuticular protein function in complex biological systems.

## Graphical abstract

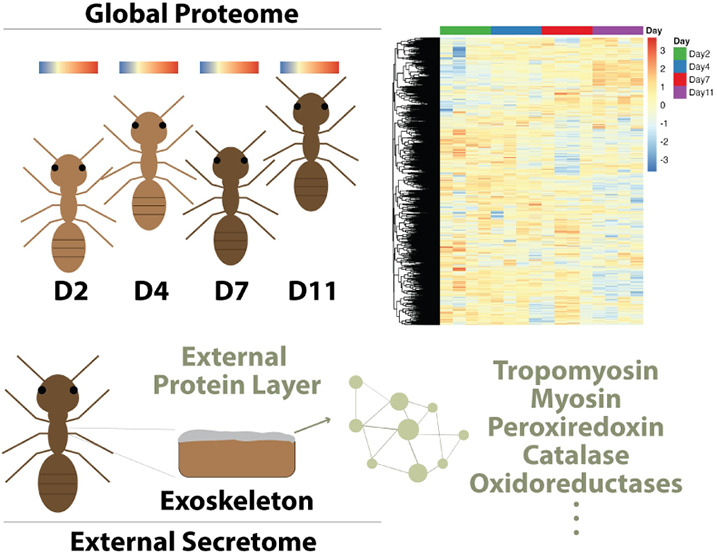

## Introduction

Proteome-wide analysis, which gives a comprehensive view of proteins expressed by an organism, offers insights into the molecular mechanisms underpinning important biological processes.^1^ While broad proteomic analysis allows for the quantification of relative protein abundance across samples, which directly reflects their contribution to biological functions,^2^ profiling the proteome temporally across developmental stages can further reveal how protein levels dynamically shift to support growth, differentiation, and adaptation over critical time points in the life of an organism.^3^ Global proteomic analyses can inform on broad-scale protein networks operating within an organism; however, they lack the spatial information required to investigate localized processes that are executed by a smaller group of proteins. The secretome, for example, is a subset of the global proteome that is secreted extracellularly and has been found to play crucial roles in local and systemic signaling, immune response, and environmental interactions.^4–7^ While mammalian secretomes have been extensively studied, revealing insights into disease pathology and therapeutic targets,^6,8,9^ secretomes in other animal groups remain relatively unexplored.

Insects, with their immense biodiversity and potential for antibiotic and therapeutic molecule discovery,^10,11^ represent an underutilized resource for investigation. Most insect secretome studies have focused on the fruit fly model organism *Drosophila melanogaster*, primarily examining internal cellular secretion processes.^12–14^ However, insect external secretomes, the proteins excreted beyond epithelial and cuticular boundaries, remain largely uncharacterized. This external proteome directly interacts with harsh biotic and abiotic environmental factors, and its composition likely reflects these external pressures. However, comprehensive profiling of cuticle-associated proteins is technically challenging, as they are often low in abundance, extensively cross-linked, or embedded within insoluble structural matrices that hinder extraction using conventional approaches.^15,16^ Thus, developing robust and transferable methods for extracting and characterizing these surface-associated proteins is crucial to uncovering how they mediate protective and defensive functions that are central to insect survival.

The leaf-cutter ant *Acromyrmex echinatior* provides a compelling system for studying the external secretome, as its cuticular surface serves as the site for diverse biological and physiological processes. These ants grow and feed a mutualistic *Pseudonocardia* bacteria on their exoskeleton, which produces antibiotic compounds that inhibit the growth of harmful colony pathogens.^17–20^ Following eclosion, newly hatched ants (“callows”) are groomed by nest-mates and inoculated with *Pseudonocardia*.^21^ A few days after inoculation, the *Pseudonocardia* exhibit an exponential growth phase, and at around 2 weeks of age, the ant is completely covered by the filamentous bacteria.^22^ This precise temporal and spatial colonization likely requires the ants to modulate bacterial growth conditions given the risk of infection from undesired entomopathogens. In addition to the beneficial *Pseudonocardia* bacteria, recent work has found that the ant’s exoskeleton is also covered by a proteinaceous layer. Li et al. (2020) detected the presence of a protein layer on the exoskeleton using hydrolysis experiments and X-ray absorption near-edge structure (XANES) spectroscopy. In this same study, *in vitro* synthesis experiments provided evidence that this extra-cuticular protein layer contributes to the formation of a Mg-calcite biomineral. This biomineral, produced *in vivo* by *A. echinatior* major ants but not minor ants, begins forming on the exoskeleton 6 days after eclosion and eventually covers nearly the entire ant body.^23,24^ Although the exact temporal dynamics of extra-cuticular protein layer secretion remains unknown, Li et al. showed that it is absent from the cuticle of newly eclosed ants and therefore likely begins to be secreted sometime during the first 6 days of adulthood.^23,24^ While its role in biomineralization is supported, it remains unclear whether the extra-cuticular proteinaceous layer also serves additional functions, such as modulating bacterial growth.

Deciphering the intricate protein networks that govern complex biological systems presents several significant challenges, such as demanding high-throughput capabilities to characterize thousands of proteins simultaneously.^15,25–29^ Over decades, mass spectrometry (MS) has become a widely adopted tool for molecular characterization and quantification due to its high sensitivity, untargeted capabilities, and high-throughput potential.^30,31^ While data-dependent acquisition (DDA) and chemical tagging strategies reliably offer accurate peptide identifications and quantifications across multiple biological samples, they pose certain limitations. DDA often introduces bias across different LC-MS runs, favoring the selection of high-abundance MS1 precursors for MS2 fragmentation due to the limited duty cycle for MS acquisition.^32^ The inconsistent precursor selections across LC-MS runs may also introduce quantitative bias. Similarly, chemical tagging, despite its accuracy and multiplexing efficiency in peptide quantification, frequently results in sample loss and reduces the level of protein identification, particularly for those of lower abundance. To address these challenges, prior studies employed a label-free data-independent acquisition (DIA) method using diaPASEF (parallel accumulation serial fragmentation). This approach adds another ion-mobility dimension, mitigates bias by avoiding fragmentation based on peak intensities, and ensures coverage of low-abundance peaks.^32–37^ The MS and ion-mobility dimensions in each diaPASEF cycle improve protein identification without compromising the quantification accuracy in proteome-wide profiling.

In this study, we utilized the diaPASEF method to quantify the whole-body proteome across developmental ages of early adult *A. echinatior* ants, representing the first developmental proteome in this species. Coupling with Gene Ontology (GO) term analysis, we identified numerous proteins with significant alterations across developmental time points. We then developed a technique to selectively isolate the micrometer-thick external secretome proteins attached to the exoskeleton, which were subsequently characterized using ddaPASEF.^38,39^ Combining this localized secretome data set with the proteome-wide analysis, we identified secreted proteins which likely play crucial roles in the early adult stage for ants. Lastly, we used STRINGdb to map protein–protein interactions of identified proteins of interest, revealing a large secreted protein network with a potential role during this critical biological window.

## Experimental Section

### *A. echinatior* Sample Preparation for Proteome-Wide Profiling

Four groups of leaf-cutter ants (2, 4, 7, and 11 days post-eclosion) with four biological replicates for each group were processed with the following procedure. Each ant was first dissolved in 150 μL extraction solution (8 M urea, 50 mM Tris buffer (pH = 8) containing 5 mM CaCl_2_, 20 mM NaCl, EDTA-free protease inhibitor, and phosphatase inhibitor) and sonicated on ice using a probe sonicator (Thermo Fisher Scientific, 50% amplitude, 15 s with a 5 s pause for 3 min and repeated for 5 cycles). After sonication, each sample was centrifuged at 14,000 × *g* for 10 min at 4 °C, and the supernatant was transferred to a new tube. The supernatant of each sample was measured by the protein bicinchoninic acid (BCA) assay to acquire the protein concentration. A 50 μg protein of each sample was aliquoted, reduced with 5 mM dithiothreitol (DTT) at 37 °C for 30 min, alkylated with 15 mM iodoacetamide (IAA) in dark for 45 min, and quenched with 5 mM DTT for 10 min. Samples were diluted with 50 mM Tris buffer to a urea concentration of <1 M. In solution protein, digestion was performed with Trypsin/Lys-C Mix (Promega) in a 50:1 ratio (protein:enzyme; w/w) at 37 °C overnight. The digestion solutions were quenched with 10% trifluoroacetic acid (TFA) to reach a final concentration of 1% TFA. Peptides were desalted with Sep-Pak C18 cartridges, dried in vacuo, and reconstituted to a concentration of 100 ng/μL peptides in 0.1% formic acid (FA) water before being analyzed by LC-MS/MS. Protein and peptide concentrations were either measured by BCA assay or NanoDrop (Thermo Scientific).

### *A. echinatior* Sample Preparation for Major and Minor Ant Comparison

Major and minor *A. echinatior* ants were collected and distinguished by body size and head width, with ants with head width >1.5 mm being grouped as majors and ants with head width <1.0 mm being grouped as minors. The outer protein layer extraction method was first optimized with different immersion times as mentioned in the Supporting Information. Each ant was immersed in 150 μL 0.1 N HCl for given time periods (0 h, 2 h, 4 h, 7 h, and 10 h with vortex and sonication) followed by neutralization with Tris buffer to pH 8 and a final volume of 1 mL. The protein extracts were collected to new tubes for proteomic sample preparation, and the remaining ant bodies were immersed in methanol until taken for SEM studies. After validating the protein layer extraction method, two sets (immersion for either 3 or 5 h) of major and minor ants were taken for the extraction with four biological replicates of each group with a total of eight leaf-cutter ants. The protein digestion was performed as described above with the difference of using C18 Ziptip to desalt samples (OMIX Tips A57003100, Waters).

### LC-MS/MS Analysis

200 ng peptides of each sample were separated on a 25 cm UHPLC column (IonOpticks, Aurora Series Emitter Column, AUR2–25075C18A-CSI, 25 cm × 75 μm, 1.6 μm C18, with a nanoZero fitting) and analyzed on an ACQUITY UPLC M-Class system (Waters, Milford, MA, USA) coupled to a TimsTOF flex MALDI 2 mass spectrometer (Bruker Scientific, LLC, Bremen, Germany). Mobile phase A was composed of optima grade water with 0.1% FA, while mobile phase B was composed of ACN with 0.1% FA. LC separation was achieved via a 120 min gradient for both diaPASEF and ddaPASEF methods at a flow rate of 300 nL/min at 50 °C: 0–95 min, 2.5–25%B; 95–100 min, 25–50%B; 100–105 min, 50–95%B; 105–120 min, 95–2.5%B. The mass spectrometer was set at *m*/*z* 100–1700 in diaPASEF mode, in which the TIMS cell was set at 0.60–1.60 V·s/cm^2^. Capillary voltage was set at 1600 V, and the collision energy was ramped linearly as a function of the mobility from 20 eV at 1/K0 = 0.6 V·s/cm^2^ to 59 eV at 1/K0 = 1.6 V·s/cm^2^. The diaPASEF window scheme^33,34,40,41^ was ranging in dimension *m*/*z* from 345.0 to 1217.0 and in dimension 1/K0 from 0.7 to 1.40 V·s/cm^2^, with 34.5 × 26 Th windows with a ramp time of 100 ms at a total cycle time of 1.59 s. The ddaPASEF method was set as follows: *m*/*z* 100–1700, a capillary voltage of 1600 V, a ramp time of 100 ms, 10 PASEF ramps, 0–5 charge, a total cycle time of 1.17 s, and dynamic exclusion for 0.4 min. The quadrupole isolation width was set to 2 *m*/*z* at *m*/*z* 700 and to 3 *m*/*z* at *m*/*z* 800. TIMS elution voltages were calibrated linearly to obtain the reduced ion mobility coefficients (1/K0) using three Agilent ESI-L Tuning Mix ions (*m*/*z* 622, 922, and 1,222).^42^ Each sample was acquired with two technical replicates.

### Data Analysis

Protein identification and quantification of mass spectrometry (MS) data were conducted using DIA-NN (version 1.8.1) and MaxQuant (version 2.5.1.0).^41,43–46^ All raw .d files were searched against the UniProt *Acromyrmex echinatior* database (Taxonomy ID: 103372, retrieved on August 26, 2023).^47^

## Results and Discussions

### Systematic Scheme of This Study

The overview scheme for this study is illustrated in Scheme 1 and addresses the following two core aims: 1) to temporally characterize the dynamics of whole-body proteomes in young adult major leaf-cutter ants (*Acromyrmex echinatior*) and obtain a comprehensive proteomic landscape and 2) to identify the components of the extra-cuticular proteinaceous layer secreted on their exoskeleton. We utilized both diaPASEF and ddaPASEF, along with a novel extra-cuticular protein isolation method, to achieve these objectives. A comparative analysis using this dual-layered approach generated a comprehensive list of external secretome-enriched proteins that also exhibited significant quantitative changes during the early adult stages of the ants. By further analyzing GO term enrichment and PPI network analyses, we shed light on the critical two-week time window during which many important local and global processes unfold in the young adult leaf-cutter ant, including metal enrichment (in the mandibles and cuticular mineral layer), modulation of native and non-native bacterial growth on the exoskeleton, cuticle sclerotization, and behavioral changes associated with shifting colony roles.

**Scheme 1:**
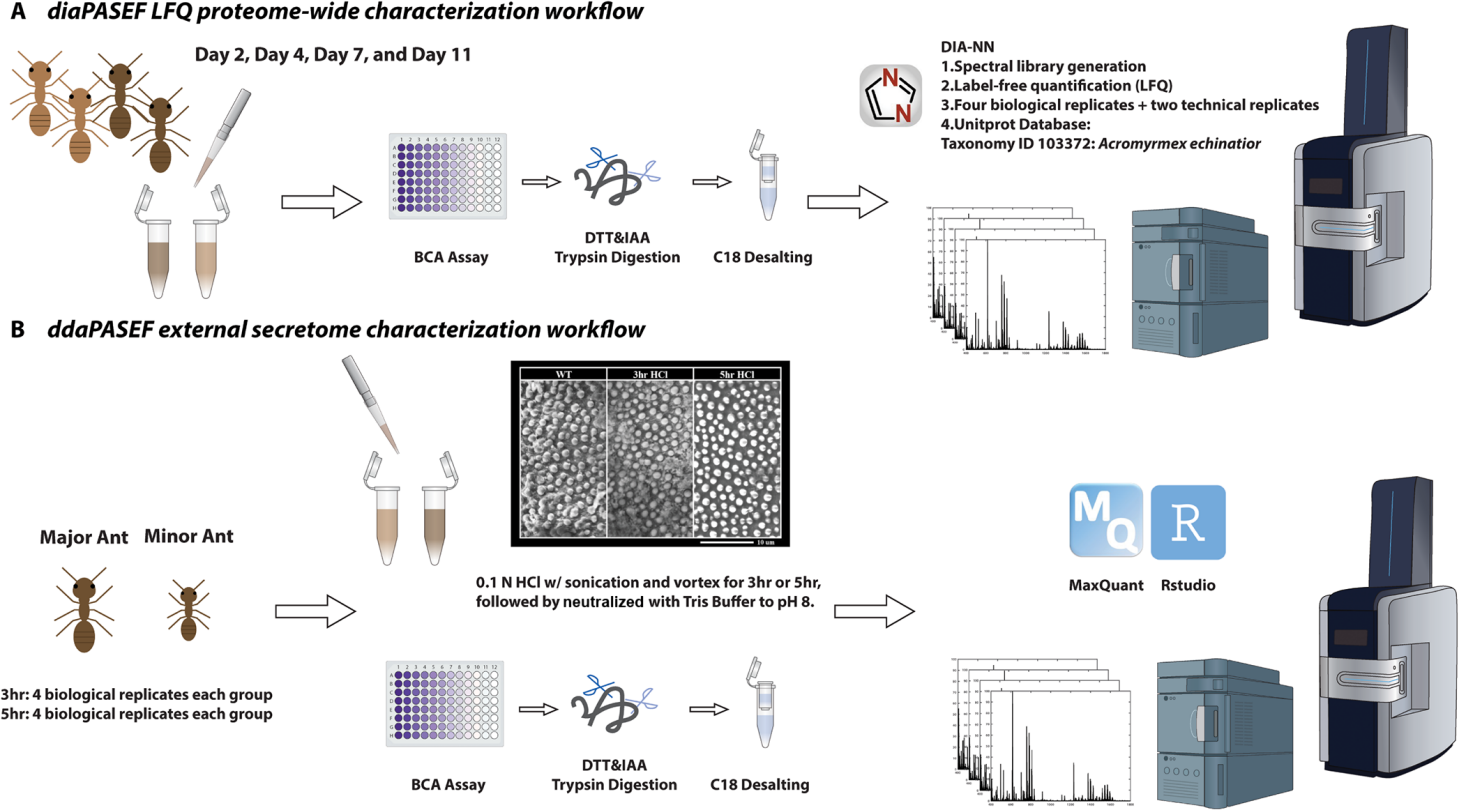
Overview of diaPASEF and ddaPASEF Workflows for Proteomic Analysis in *A. echinatior* ^a^

a (A) The diaPASEF LFQ proteome-wide analysis workflow. (B) The surface protein isolation for ddaPASEF secretome characterization workflow.

### Proteome-Wide Characterization across *A. echinatior* Young Adult Development

To quantitatively analyze whole-body proteome alterations across early adult development, we first employed diaPASEF on four ages of *A. echinatior*, sampled at 2, 4, 7, and 11 days post-eclosion (Scheme 1A). These 120 min diaPASEF runs quantified 4,428 proteins across all groups (Figure 1A and Supplementary Data 1—Proteome). Of these, 1,347 proteins showed statistically significant differences in their quantities between the four groups (*p*-value < 0.05) based on one-way ANOVA. The 1,347 significantly changed proteins were further subjected to hierarchical *k*-means clustering, which resulted in four distinct clusters (Figure 1B and Supplementary Data 1—Cluster), illustrating distinct patterns of quantitative alteration across developmental ages. In general, we discovered that the proteins in clusters 1 and 2 increased across the age groups, while clusters 3 and 4 showed decreasing trends (Figure 1C).

**Figure 1:**
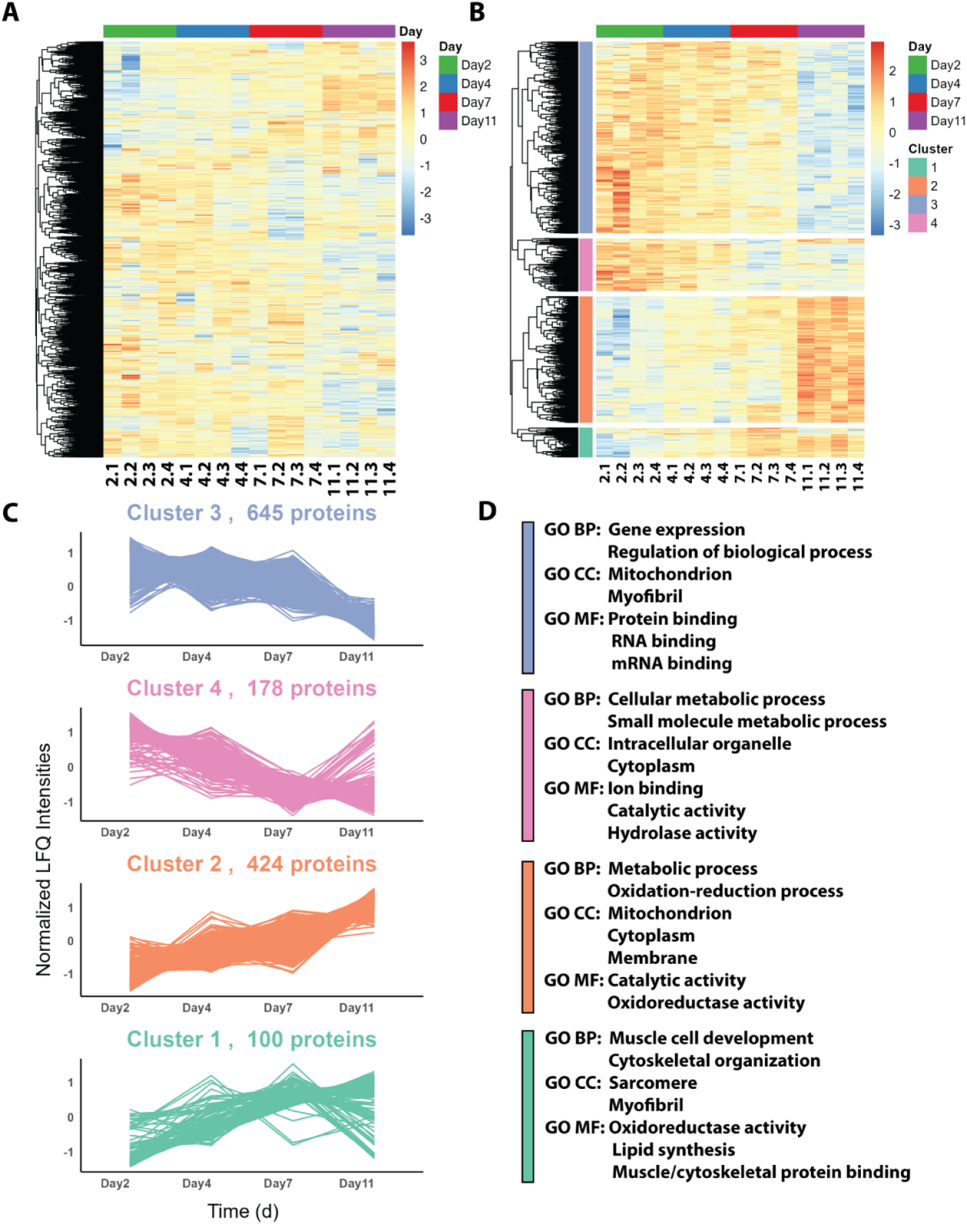
Clustering and functional enrichment analysis of proteomic data across different developmental ages in *A. echinatior*. Samples were labeled with “2.1” to “11.4”, representing Day 2 sample #1 to Day 11 sample #4, respectively. (A) Heatmap displaying the hierarchical clustering of proteins identified across four developmental ages. (B) Clustering revealed four distinct clusters, with the number of proteins in each cluster indicated. The normalized LFQ intensities for each cluster show the dynamic changes in protein abundance over time. (C) Quantitative plots for each cluster. (D) Functional enrichment analysis for each cluster, showing significant top biological processes, cellular components, and molecular functions associated with the proteins within each cluster.

To further elucidate the biological significance of each cluster, we conducted Gene Ontology (GO) enrichment analyses, identifying the top 10 pathways for “Biological Process (BP),” “Cellular Component (CC),” and “Molecular Function (MF)” (Figures 1D and S1). Cluster 1, which generally consists of proteins that increase in abundance from day 2 through day 7 and then either plateau or decrease at day 11, consisted of 100 proteins primarily associated with muscle cell development, cytoskeletal organization, and related molecular functions (Figure S1—Cluster 1/BP). This cluster is also enriched in lipid synthesis pathways (e.g., prostanoid receptor and prostaglandin receptor) and muscle/cytoskeletal protein binding (e.g., tropomyosin and actin). Notably, over 50 proteins in this cluster are involved in oxidation–reduction processes (Figure S1—Cluster 1/MF). Cluster 2, comprising 424 proteins, exhibited a consistent increase across all four developmental ages. This cluster was enriched in energy-related proteins, particularly those associated with mitochondria, as well as metabolic pathways, indicating high activity in energy production and metabolic processes. On the other hand, clusters 3 and 4, containing 645 and 178 proteins, respectively, generally consisted of proteins that decreased in abundance throughout the sampled developmental ages, although cluster 4 proteins plateaued or increased in abundance on day 11. Both clusters 3 and 4 were enriched for GO terms associated with reductions in gene expression, the regulation of biological processes, and small-molecule metabolic activities. In terms of molecular function, proteins related to protein, RNA, mRNA, and ion binding were enriched in the clusters. Overall, the cluster-wise GO enrichment analyses provided a comprehensive understanding of each cluster within this extensive proteome data set, offering insights into the biological and molecular functions of proteins that are highly abundant in the early adult life of *A. echinatior* major worker ants.

To further stratify proteins significantly altered across developmental ages, we regrouped the four developmental ages into “callow” (Day 2 and Day 4) and “young adult” (Day 7 and Day 11) (Figures 2 and S2) representing newly eclosed ants and young adult ants with a proteinaceous layer. We identified 89 and 92 proteins exhibiting higher detected abundance in the callow and young adult groups, respectively. GO enrichment analysis of the proteins exhibiting higher detected abundance in callow workers revealed the information from UniProt annotations with predicted localization in extracellular regions. Many BP terms were related to glycoside, amino sugar, and aminoglycan metabolic/catabolic pathways, likely due to the chitinization of the exoskeleton at the callow worker stage, with chitin being a long-chain polymer of N-acetylglucosamine and the primary component of the ant cuticle.^48^ We further evaluated GO BP results in callow workers with network term analysis, which revealed networks corresponding to “Response to fungus” and “Cell killing” of other organisms, including several Major Royal Jelly Proteins (MRJPs) (Figure S3A). MRJPs, also known as bee-milk proteins, have been previously associated with caste development and sex determination.^49^ This finding is significant as many important *A. echinatior* colony tasks are caste-specific. Additionally, UniProt annotations indicate that many callow-enriched proteins contain domains involved in disulfide-bond formation or are categorized as structural constituents of the cuticle, consistent with early cuticle stabilization processes during the callow stage (Figure S2A).^50,51^

**Figure 2:**
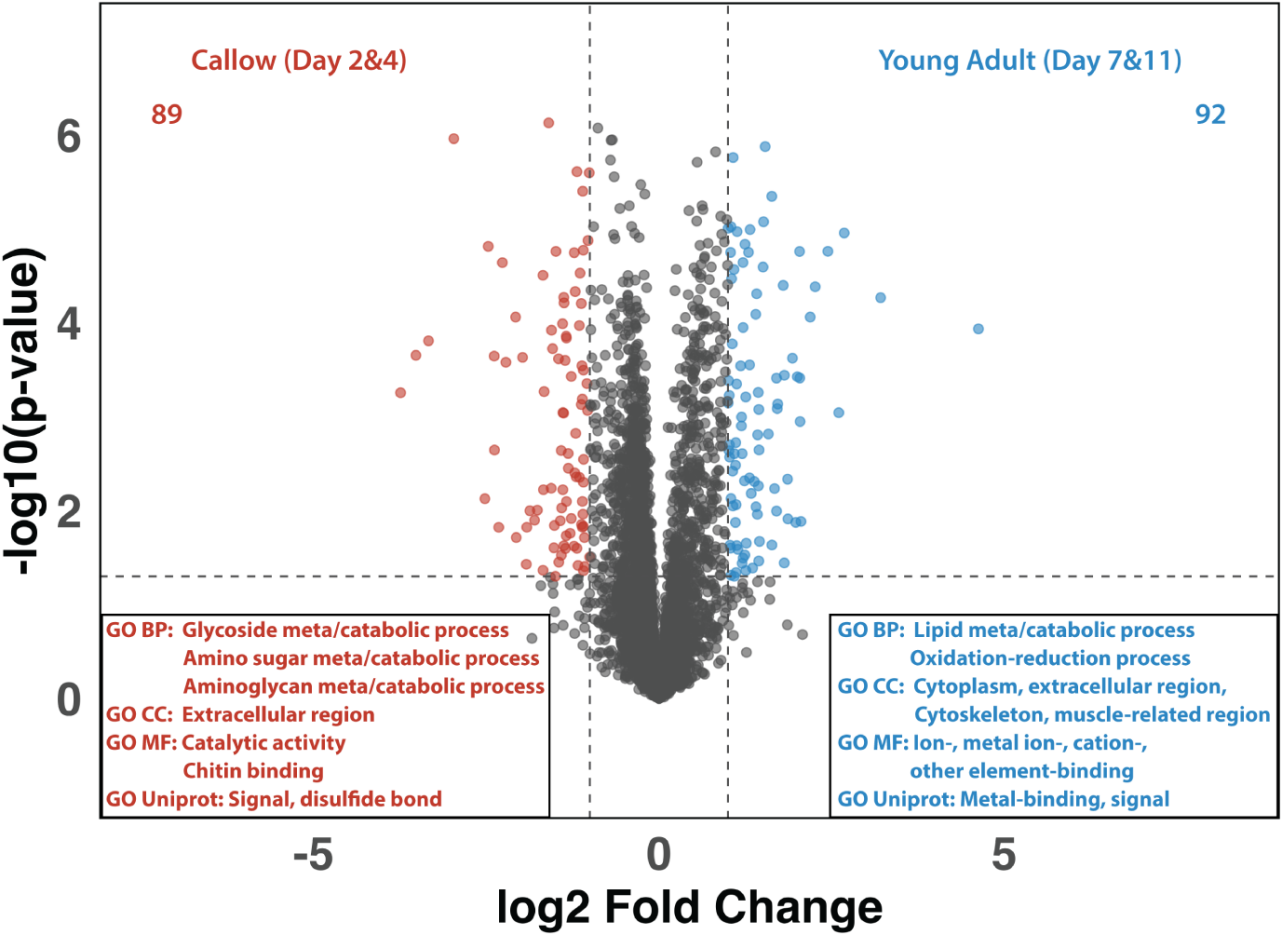
Volcano plot and GO analysis of the proteomes of callow (days 2 and 4) vs young adult (days 7 and 11) *A. echinatior* workers. The volcano plot compares protein expression between callow and young adult workers, illustrating proteins with significantly higher abundances in callow (left, red) and young adult (right, blue) ants. GO analysis of proteins with higher abundance in callows reveals significant enrichment in metabolic processes, extracellular region, and catalytic activities, emphasizing chitin catabolic processes and cuticle development (red). GO analysis of proteins with higher abundance in young adults highlights processes related to muscle structure development, oxidoreductase activity, and iron binding, suggesting the importance of muscle cells in maintaining structural integrity (blue).

In the young adult group, proteins exhibiting higher detected abundance were enriched with GO terms associated with cellular components, including the cytoplasm, extracellular region, cytoskeleton, and muscle-related regions (Figure 2). Many biological processes were linked to lipid metabolic and catabolic activities, suggesting that young adult workers exhibit more enriched and active lipid/fatty acid processing pathways compared to callow worker ants. Furthermore, we performed the GO term network analysis, which resulted in the clustering of lipid/fatty acid metabolism and energy-related molecular functions for young adult workers, including eicosanoid receptor activities responding to eicosanoid signaling molecules and NAD binding associated with redox and biosynthetic reactions (Figure S4C). Additionally, a large cluster of MF terms related to metal ion-, ion-, cation-, and other element-binding interactions was identified, which was corroborated by UniProt annotations (Figure S4D). These findings are interesting, considering that various metal-enriching processes occur in young major workers, including Mg-enriched calcite formation and Zn-enrichment of the mandibles.^24,52^

### Surface Protein Isolation for Characterization of the External Secretome of Major Worker Ants

Next, to characterize the composition of the external secretome of *A. echinatior* ants, we aimed to develop a method to isolate this protein layer from the cuticular surface for downstream LC-MS/MS analyses. This extra-cuticular proteinaceous layer was recently reported in mature *A. echinatior* major ants,^24^ which we confirmed with scanning electron microscopy (SEM) analysis (Figure S5C). In comparison, we also observed that minor ants do not have a visible proteinaceous layer and therefore serve as ideal comparative control. To optimize the isolation of this secreted proteome, we subjected major worker ants to HCl treatment for varying durations of 2, 4, 7, and 10 h. SEM indicated substantial removal of the protein layer from major workers following 4 h of treatment, with near-complete removal after 7 h (Supporting Information—Method and Figure S5A).

After achieving the proof of concept, we systematically applied this surface protein extraction method on the major and minor workers with biological replicates using 3 and 5 h extraction. Protein BCA assays corroborated this method’s efficacy, showing an increase in protein concentration from 3 h to 5 h in major workers (Supplemental Data 1—Extrac_first_time|major minor). Additionally, the LC-ddaPASEF-MS/MS proteomic profiling identified 61 and 323 proteins from the 3 h- and 5 h-treated major worker samples, respectively. To validate that the characterized proteins originated from the externally secreted protein layer, we treated minor ants with HCl for 5 h and characterized the extracted proteins as a comparative reference. This resulted in the identification of only 14 proteins, compared to the 323 of similarly treated major ants. Furthermore, the BCA assay and mass spectrometry of minor worker extracts yielded negligible results. SEM imaging provided a direct visual comparison of the cuticular surfaces of major and minor workers subjected to the extraction procedure (Figure S5C), confirming that HCl treatment successfully removed surface proteins from major ants without causing significant mechanical disruption to the cuticular surfaces of either major or minor ants. While this validation strongly supports our method’s specificity, we acknowledge that acid extraction inherently introduces limitations, such as a solubility bias toward acid-soluble proteins and a potential risk to protein integrity, even though our high identification rate confirms the method’s effectiveness for broad-scale characterization. Given that the 5-h treatment proved sufficient for robust extraction of the secretome layer without significant denaturation, we further analyzed the isolated external secretome of 5 h HCl-treated major ants.

To identify proteins potentially secreted extra-cuticularly and temporally altered in abundance at different ages during the early adult stages of major ants, we examined the overlap between the 323 proteins identified by HCl extraction and the 1347 proteins significantly altered in abundance at different ages in our prior whole-body proteome characterization (Figure S10). The intersection of these two data sets yielded a list of 175 proteins. To gain insight into the diversity and complexity of these surface proteins, we visualized the GO-term network for biological processes (BP) (Figure 3C). This network revealed nine distinct clusters, each representing different categories of biological functions. For example, one resulting cluster highlights muscle system processes, suggesting that a significant portion of surface proteins is involved in muscle cell development, cytoskeleton structure organization, tissue formation, and muscle contraction. Another cluster represents functions related to responses to environmental stimuli and interaction with other organisms, likely crucial for communication and social interaction. Terms for the active catabolism of biomolecules, such as organic acids and organonitrogen compounds, also clustered together. The largest cluster, although not associated with a single common biological process, comprises a range of metabolic functions, including carbohydrate metabolism/biosynthesis, energy production (TCA cycle and cellular respiration), lipid and fatty acid metabolism/catabolism, amino acid biosynthesis, and metabolism/catabolism of various biomolecules. This network analysis underscores the complexity of the biological processes occurring on the surface of the exoskeleton, revealing the dynamics of a diverse array of secreted biomolecules on the exoskeleton of these ants. Furthermore, we have evaluated the surface-associated proteins using DeepLoc 2.1 to assess the presence of signal peptides and predicted subcellular localization as part of the secretome quality check (Figures S13 and S15).^53,54^

**Figure 3:**
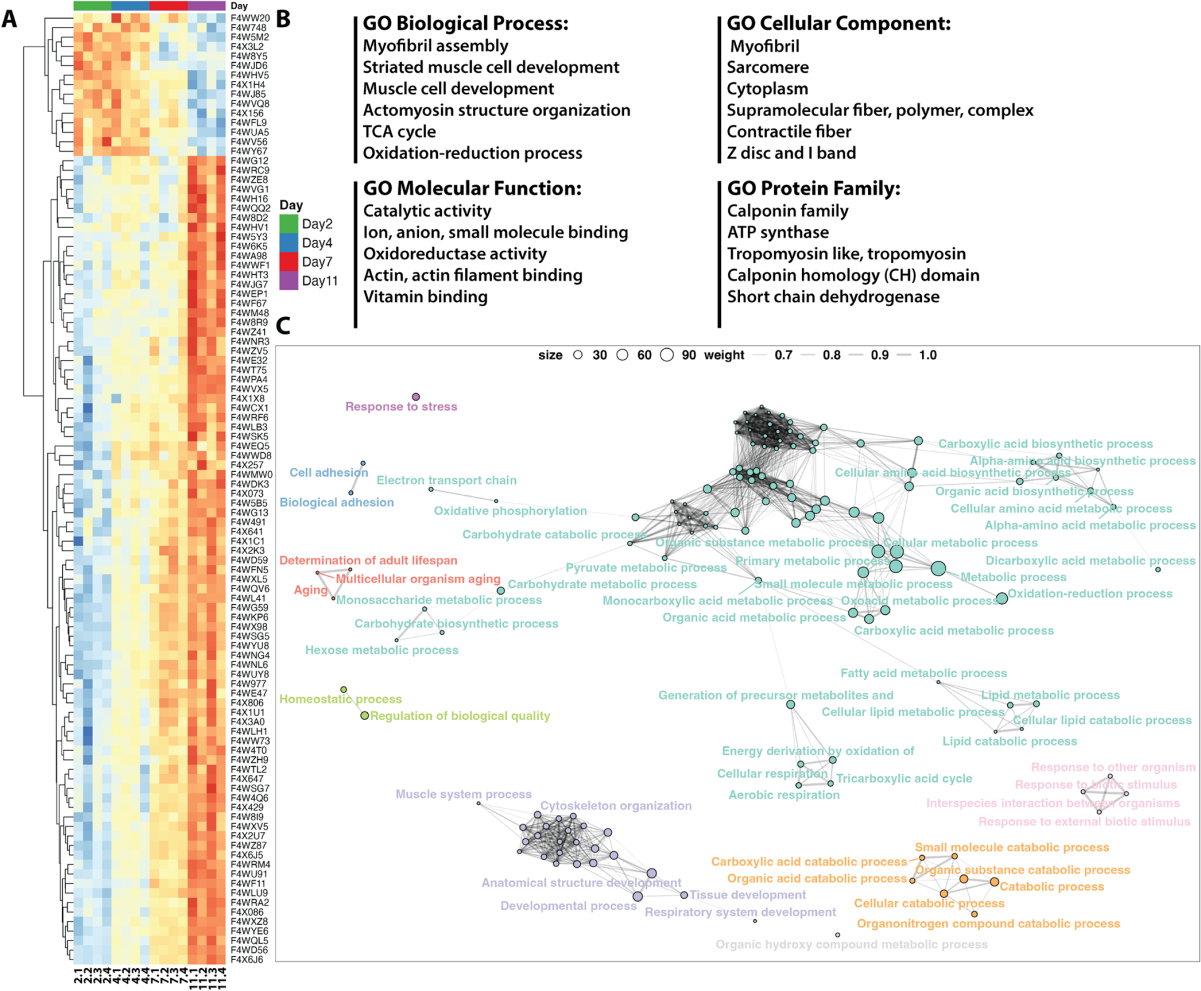
Secretome characterization and GO term networks in *A. echinatior*. (A) Heatmap showing proteins with significant temporal alterations in the whole-body proteome that were also identified in the external secretome layer. Proteins are clustered based on their abundance across different time points. (B) GO enrichment analysis of external secretome proteins. (C) Network visualization of interconnected GO terms associated with biological processes (BP), with each color representing a different functional category (purple = muscle organization system; pink = response to other organisms; orange = catabolism; mint green = energy production/metabolism; red = aging; light green = homeostasis; magenta = stress response).

Given that the callow and young adult ant stages are critical time points for colonization and growth of beneficial *Pseudonocardia* bacteria on the ant exoskeleton, we further explored clusters related to environmental stimuli, including the “Response to other organism” and “Response to stress” networks (Figures S11–16). The “Response to other organisms” cluster contained 4 nodes composed of major proteins such as peroxiredoxin, transferrin, laminin, and flotillin. In this cluster, we identified three peroxiredoxin proteins, which have predicted antioxidation activities and are known to protect insects from reactive oxygen species (ROSs) and other reactive species (Figure S11). Transferrin, a protein involved in iron metabolism and transport to reduce oxidative stress and inhibit microbial growth, was also identified under the same cluster (Figure S12).^55^ Of note, the peroxiredoxin and transferrin proteins showed increasing abundance across the early adult stages (Figures S12 and S14). Additionally, laminins, neuroglia, and flotillin proteins, which are integral to cellular architecture, signaling, and intercellular communication, were also found to be present under this “Response to other organisms” cluster; however, these proteins generally were found to decrease in abundance across development. On the other hand, the “Response to stress” cluster contained a single node, composed of two proteins (Figure S16). One protein was a ″protein lethal essential for life”, a member of the heat shock 20 protein family. The other protein was catalase, which is an enzyme that catalyzes the reduction of hydrogen peroxide. Prior studies have shown that high concentrations of hydrogen peroxide during calcium carbonate formation can affect crystal morphology,^56^ potentially linking our finding with the *A. echinatior* biomineralization process.

Maintaining a symbiotic relationship with native bacteria like *Pseudonocardia* requires modulating growth conditions that support colonization of the native symbiont while suppressing the growth of pathogens. This makes proteins such as transferrins particularly intriguing, as they have been shown to contribute to microbial defense through iron sequestration.^55,57^ We found F4W957 transferrin enriched in the secretome and exhibiting an increasing abundance over time in the whole-body proteome analysis (Figure S12). Interestingly, the temporal increase of this protein correlates with increasing *Pseudonocardia* growth on the ant cuticle over the same period. This suggests a potential mechanism to reduce the growth of unwanted bacteria on the exoskeleton, thereby minimizing competition for their microbial symbionts. However, direct biological validation is needed to determine whether these abundance dynamics are functionally linked to symbiont colonization or the suppression of competing pathogens. Nevertheless, by enabling characterization of age-dependent whole-body proteomes and surface-associated proteins, our method provides insights into host–symbiont interactions and developmental immune modulation in *A. echinatior*.

To further refine our characterization of important external secretome proteins, we applied a more stringent filter (*p*-value <0.001) across developmental ages. This resulted in 100 significant proteins (Figure 3A), which were then used for GO enrichment analysis (Figures 3B and S17). Notably, among these 100 proteins identified, most were associated with clusters 1 and 2 from our whole-body proteome results, which displayed increasing trends across the four developmental ages (16 and 70 proteins, Figure S18). The GO enrichment analysis of BP highlighted muscle cell-related developmental processes—such as myofibril assembly, striated muscle cell development, muscle cell development, and actomyosin structure organization—among the top 10 pathways, along with energy-related pathways like the TCA cycle (Figures 3B and S17). The CC also revealed a predominance of muscle and cytoskeleton-related components, excluding mitochondria, in the top pathways. Meanwhile, MF terms related to anion and ion binding were enriched, suggesting a possible link to the incorporation of Mg^2+^ and Ca^2+^ during the biomineralization process of this ant species.

### Protein–Protein Interaction (PPI) Network Identified in STRINGdb

Insects possess a highly ordered chitin-based extracellular matrix that forms the cuticle, functioning not only as a protective barrier but also as a biomechanical interface linking epidermal cells, tendons, and muscles. The layered procuticle, with its horizontal laminae and vertical pore canals, provides structural rigidity and serves as a substrate for muscle attachment through tendon cells.^58^ Recent work further highlights that specialized tendon (apodeme) epithelial cells secrete chitinous internal cuticle at muscle attachment sites, where integrins, ZP-domain proteins, and microtubule networks mediate force transmission from muscle to cuticle.^60^ Disruption of chitin-modifying enzymes leads to tendon-cuticle failure and muscle detachment, underscoring the functional integration between extracellular matrix composition and locomotor mechanics. These studies suggest that cuticle-associated secretomes can contain not only classical cuticular proteins but also muscle-linked components involved in adhesion and force transmission. Consistent with this, our surface secretome and proteome-wide analysis revealed enrichment of muscle-associated proteins at specific developmental stages, supporting the view that the insect cuticle–tendon–muscle continuum is dynamically remodeled and that extracellular proteins originating from musculature or tendon cells may be detected on the cuticle surface during periods of active growth and biomechanical restructuring. With the consistent observation of muscle-related proteins in both the whole-body proteome and external secretome data, we next performed protein family (Pfam) enrichment analysis to identify the essential protein families present in the secretome (Figure 3B—Protein family). These results indicated that several protein families, particularly those related to tropomyosin and tropomyosin-like proteins (e.g., Calponin family repeat, tropomyosin, and calponin homology (CH) domain), were highly enriched in this layer.

To further investigate the interactions of the significantly enriched tropomyosin-family proteins, we utilized STRINGdb to map the predicted protein association network of this family. STRINGdb integrates evidence from experimental data, curated annotations, and coexpression information for depicting potential functional relationships among protein networks. Therefore, STRINGdb serves as a powerful bioinformatic tool to visualize functional associations among identified proteins within each cluster and to confirm that proteins grouped by GO term enrichment also exhibit coherent associations at the network level.^61^ This resulted in a PPI network containing 31 proteins (Figure 4A). Out of these 31 proteins, 18 proteins were present in both our isolated secretome and whole-body proteome profiling experiments. To gain further insight, we examined their quantitative alterations across different developmental ages by revisiting the whole-body proteome data (Figure 4B), finding that most tropomyosin-related proteins exhibited increasing trends, while myosin-related proteins showed opposing trends. Remarkably, three secretome-enriched tropomyosin proteins—F4WSG5 Tropomyosin, F4WSG7 Tropomyosin, and F4X196 Tropomyosin-1—clustered within the PPI network, suggesting strong interactions and communication between these proteins. Intriguingly, across all four developmental ages, tropomyosin-family proteins, in addition to other secretome proteins, consistently ranked among the top proteins in terms of LFQ values, signifying the presence of secretome proteins in the crucial stages of *A. echinatior* development. Notably, after Day 7, seven proteins within this PPI network ascended into the top 25 most abundant proteins (Figure S19, highlighted in red) out of the 1,347 significantly changed proteins.

**Figure 4:**
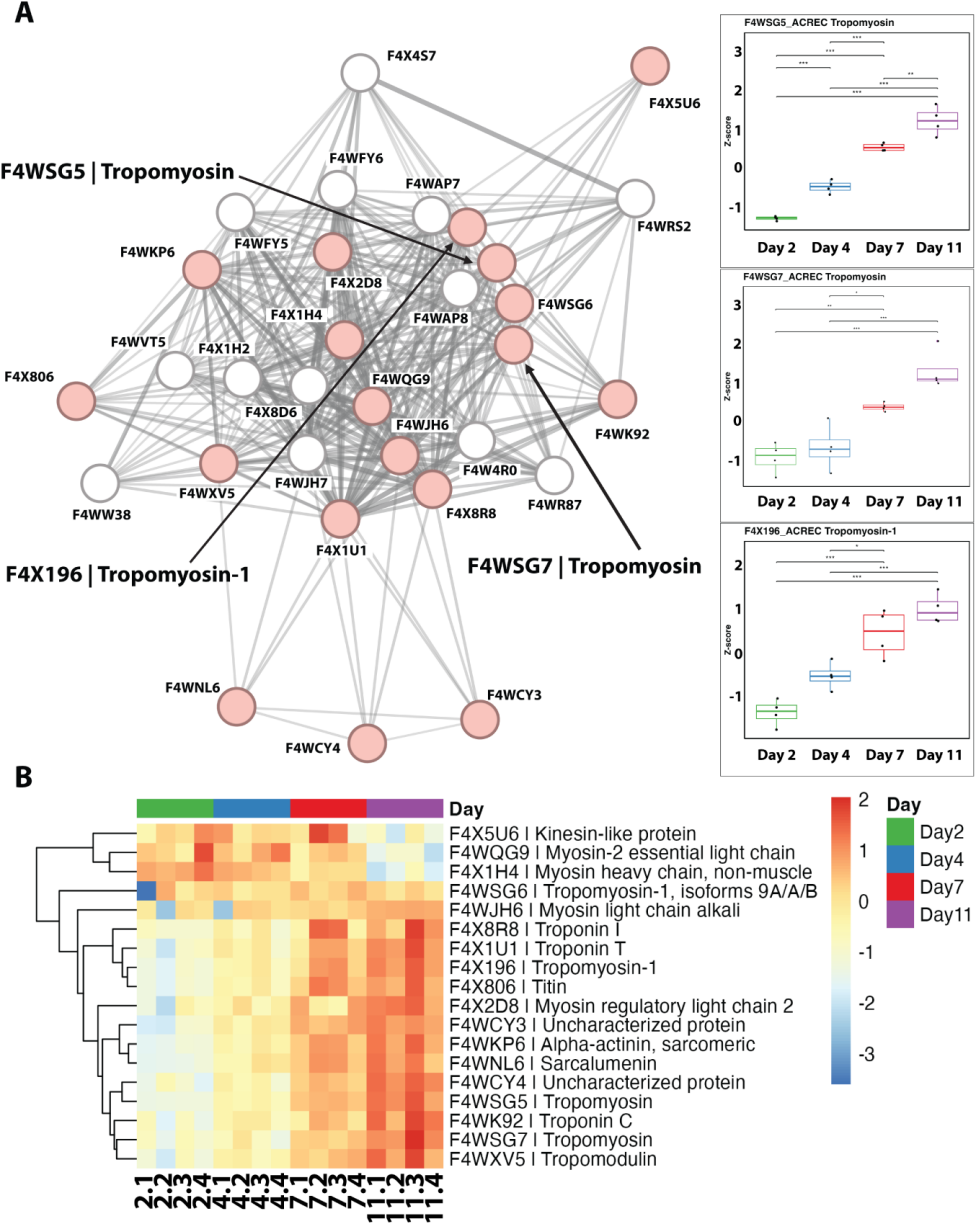
Protein–protein interaction (PPI) network and associated quantitative changes across developmental ages. (A) STRING PPI network for key muscle-related proteins, such as tropomyosin and titin, identified in the proteome of *A. echinatior*. Box plots show significant temporal changes in protein abundance based on whole-body proteome results. Among the 31 proteins in the PPI network, three tropomyosin proteins—F4WSG5 Tropomyosin, F4WSG7 Tropomyosin, and F4X196 Tropomyosin-1—clustered within this network, all of which were present in the external protein layer. Of the 31 proteins in this network, 18 were localized to the external secretome layer of *A. echinatior* (colored in red). (B) Heatmap depicting quantitative changes in the PPI proteins enriched in the STRING network shown in (A) across days 2, 4, 7, and 11.

Tropomyosin, a well-known actin-regulating protein in both muscle and nonmuscle cells, plays a crucial role in muscle contraction and stabilization of the cytoskeleton.^62^ Its presence and differential expression across developmental ages suggest increased muscle development in young adult ants. This aligns with behavioral observations, as newly eclosed callow worker ants exhibit very limited movement or activity, followed by increasing mobility over time.^63^ In our results, tropomyosin was also highly enriched in the extra-cuticular secretome layer, which is an interesting connection with the previously proposed correlation between tropomyosin and Mg-calcite biomineral formation.^64^ In a recent study, sea urchin tropomyosin was found to play a role in enriching the Mg concentration of their calcareous spines. The incorporation of Mg^2+^ into calcite crystals requires a high activation energy due to the extreme interaction between Mg^2+^ and water molecules.^15,23,65^ The exact mechanism of how the sea urchin tropomyosin can induce Mg-enriched calcite remains unknown; however, it was hypothesized that the OH or COOH groups of tropomyosin could bind to Mg^2+^ and increase their reactivity with anions such as carbonate to form Mg-containing calcite. In their study, they found that the sea urchin tropomyosin contains 25.9% acidic amino acids (7.7% aspartic acid and 18.2% glutamic acid). Comparatively, we found 22.4% (6.1% aspartic acid and 16.3% glutamic acid), 16.4% (7.2% aspartic acid and 9.2% glutamic acid), and 21.4% (7.2% aspartic acid and 14.4% glutamic acid) acidic amino acids for F4WSG5, F4WSG7, and F4X196, respectively (Figures S20–S22). Our results show a comparable ratio of acidic amino acids, suggesting a similar capacity for Mg^2+^ binding. Together, this trend suggests that these proteins are not only localized in the epicuticle region but also undergo significant quantitative changes during the secretion of the extra-cuticular protein layer in *A. echinatior*, highlighting a potentially pivotal role for the tropomyosin family in the secretome and underscoring their structural and functional contributions to this critical protein layer.

## Conclusion

In this study, we present a novel dual-layered proteomic approach that differentiates between the whole-body proteome and the secretome of *Acromyrmex echinatior*, offering valuable insights into developmental processes, stress responses, and interspecies interactions. By integrating quantitative proteomics with an advanced protein isolation technique, we identified key secretome components including tropomyosin and other functional proteins associated with structural integrity, microbial defense, and stress adaptation. This methodological framework demonstrated high sensitivity and specificity in capturing surface-bound proteins, establishing it as a powerful tool for dissecting spatially localized subproteome networks. Its versatility and translatability make it broadly applicable for profiling secretomes and investigating host–microbe interactions across diverse biological systems, with potential implications for developmental biology, symbiosis, and applied proteomics.

## Supplementary Material







## Data Availability

The LC-MS/MS raw data and supplementary files have been deposited to the ProteomeXchange Consortium via the MassIVE partner repository with the accession number “MSV000098331”.

## Notes

Supporting methods and Figures S1–S22 (PDF)
Supplementary Data S1. Proteome database search results, clustered groups, volcano plot, extraction optimization results, and secretome searched against combined database results (XLSX)
Supplementary Data S2. Gene ontology enrichment results (XLSX)
