# Supplementary material for: Proteome-Wide Analysis and Surface Protein Isolation for Secretome Characterization Reveal Insights into the Biology of the Leaf-Cutter Ant Acromyrmex echinatior
Source: Anal Chem. 2025 Dec 11;97(51):28281–91. doi: 10.1021/acs.analchem.5c05220 (PMC12756850; doi:10.1021/acs.analchem.5c05220)
Supplement: Supplementary file 1 [file ac5c05220_si_001.pdf]

Supporting Information

Proteome-wide analysis and surface protein isolation for secretome characterization reveal insights into the biology of the leaf-cutter ant *Acromyrmex echinatior*

Penghsuan Huang[1,†], Joseph Sardina[2,†], Haiyan Lu[3], Gaspar Bruner-Montero[4,5,6,7], Cameron R. Currie[2,5,*], Lingjun Li[1,3,8,9,*]

[1] Department of Chemistry, University of Wisconsin-Madison, Madison, WI 53706, USA

[2] Department of Bacteriology, University of Wisconsin-Madison, Madison, WI 53706, USA

[3] School of Pharmacy, University of Wisconsin-Madison, Madison, WI 53705, USA

[4] Estación Científica Coiba-AIP, Ciudad del Saber, Clayton, Panamá 0816-02852, Panama

[5] Department of Biochemistry & Biomedical Sciences, McMaster University, Hamilton, Ontario L8S 4K1, Canada

[6] Centro de Biodiversidad y Descubrimiento de Drogas, Instituto de Investigaciones Científicas y Servicios de Alta Tecnología-AIP (INDICASAT-AIP), Ciudad del Saber, Clayton, Panamá 0843-01103, Panama

[7] Sistema Nacional de Investigación (SNI), Secretaría Nacional de Ciencia, Tecnología e Innovación (SENACYT), Ciudad del Saber, Clayton, Panamá 0816-02852, Panama

[8] Lachman Institute for Pharmaceutical Development, School of Pharmacy, University of Wisconsin–Madison, Madison, WI 53705, USA

[9] Wisconsin Center for NanoBioSystems, School of Pharmacy, University of Wisconsin–Madison, Madison, WI 53705, USA

[†]Equal Contribution: Penghsuan Huang and Joseph Sardina contributed equally to this work.

*Corresponding authors:
Cameron Currie: currie@bact.wisc.edu
Lingjun Li: lingun.li@wisc.edu

Table of Content

List of Supplementary Methods, Figures, Tables, and Data.

Extended experimental section.

Method development of deciphering surface layer proteome.

Supplementary Figure S1: GO Functional enrichment analysis for each cluster of proteome-wide analysis

Supplementary Figure S2: GO Analysis of the proteomes of callow (Day 2 and 4) vs. young adult (Day 7 and 11) *A. echinatior* workers. Volcano plot from **Figure 2**.

Supplementary Figure S3: GO term network of the proteins upregulated in the "Callow".

Supplementary Figure S4: GO term network of the proteins upregulated in the "Young Adult".

Supplementary Figure S5: SEM Validation and Protein Extraction Over Time.

Supplementary Figure S6: GO term network BP(5h, $p < 0.001$).

Supplementary Figure S7: GO term network CC(5h, $p < 0.001$).

Supplementary Figure S8: GO term network MF(5h, $p < 0.001$).

Supplementary Figure S9: GO term network Pfam(5h, $p < 0.001$).

Supplementary Figure S10: Venn diagram of the secretome & whole-body proteome IDs.

Supplementary Figure S11: Proteins categorized under the pink cluster with the biological functions of responding to external environmental stimulus.

Supplementary Figure S12: Proteins categorized under the pink cluster with the biological functions of responding to external environmental stimulus.

Supplementary Figure S13: F4W957 Transferrin as an example of bioinformatic tool DeepLoc 2.1-predicted secretome protein.

Supplementary Figure S14: Proteins categorized under the pink cluster with the biological functions of developmental adaptions.

Supplementary Figure S15: F4WY67 Laminin subunit alpha as an example of bioinformatic tool DeepLoc 2.1-predicted secretome protein.

Supplementary Figure S16: Proteins categorized under the purple cluster ("Response to stress") with the biological functions of stress response.

Supplementary Figure S17: GO Functional enrichment analysis for secretome characterization, extended data from **Figure 3B**.

Supplementary Figure S18: The Venn diagram shows the intersection of proteins extracted by HCl treatment.

Supplementary Figure S19: LFQ intensity-based comparison of protein abundance across developmental ages.

Supplementary Figures S20-22: Tropomyosin protein amino acid calculation

Supplementary Data S1 – Proteome database search results, clustered groups, volcano plot, extraction optimization results, and secretome searched against combined database results. (Separate Excel spreadsheet)

Supplementary Data S2 – Gene ontology enrichment results. (Separate Excel spreadsheet)

Chemicals and materials. Optima UPLC-grade acetonitrile (ACN), Optima UPLC grade water, Optima LC/MS grade formic acid (FA), calcium chloride dihydrate ($CaCl_2 \cdot 2H_2O$), Urea, Tris base, and hydrochloric acid (HCl) were obtained from Fisher Scientific (Hampton, NH). Iodoacetamide (IAA), trifluoroacetic acid (TFA), and dithiothreitol (DTT) were purchased from Sigma-Aldrich (St. Louis, MO). EDTA-free Protease Inhibitor Cocktail and Phosphatase Inhibitor Cocktail tablets were acquired from Roche (Basel, Switzerland). Pierce BCA protein assay kit and quantitative colorimetric peptide assay were obtained from Thermo Fisher Scientific (Waltham, MA). Mass spectrometry grade Trypsin/Lys-C were purchased from Promega (Madison, WI). Sep-Pak C18 cartridges were purchased from Waters Corporation (Milford, MA).

Scanning Electron Microscopy (SEM) Samples were removed from ethanol and dehydrated via air-drying overnight, and were then placed on a carbon adhesive stuck to an aluminum stub. Ants were not sputter-coated prior to imaging. Images were taken using a either a Zeiss LEO 1550 variable pressure SEM at 1.5 kV voltage or a Hitachi s3400N variable pressure SEM at 15 kV voltage.

Culture of *Acromyrmex echinatior* ants for temporal proteome A colony of *Acromyrmex echinatior* was collected from Gamboa, Panama and housed indoors in a plastic container. In order to monitor eclosion from the pupal to the adult stage, smaller sub-colonies were set up. Sub-colonies were housed within 150x15 mm clear petri-dishes, and consisted of ~1 g of fungal cultivar placed in a 35x15 mm clear culture dish, a water-soaked cotton ball, ten minor worker ants, 4 media worker ants, and 4 major worker ants. Pupae were then collected from the main colony, and placed in the fungal cultivar of the sub-colony. The sub-colonies were checked at the same time once per day for eclosion, and any eclosed ants were transferred to a new sub-colony as described above. The eclosed ants were then raised for the desired timepoints (2, 4, 7, or 11 days), at which point they were stored in sterile 1.5 mL centrifuge tubes at -80°C until further processing.

Extended data analysis method section

DIA-NN Search Parameters: A total of 32 diaPASEF .d files were subjected to a library-free search. The parameters included the use of Trypsin/P as the protease, allowing up to 2 missed cleavages, and a maximum of 1 variable modification per peptide. The modifications included N-terminal M excision and C-terminal carbamidomethylation. The peptide length was set between 7 and 30 amino acids, with precursor charges ranging from 1 to 4, *m/z* values from 350 to 1250, and fragment ion *m/z* values from 100 to 1700. A precursor FDR of 1.0% was applied, and both match-between-runs (MBR) and the robust LC quantification strategy were enabled.

MaxQuant Search Parameters: A total of 16 ddaPASEF .d files (comprising 4 major and 4 minor ants with 3h and 5h extractions) were analyzed. The parameters included a protein FDR of 0.01, peptide lengths of 7 to 40 amino acids, and modifications such as oxidation (M) and acetylation (protein N-terminus). The MBR feature was enabled.

After database searching, the result files were loaded into the R Studio for data post-processing and visualization. All the missing values were imputed in DIAgui with the "MinDet" method and technical replicates were averages.[1] The identified proteins were filtered by the following criteria: number of unique peptides >= 1 and Score > 40. Gene ontology (GO) enrichment analysis was performed using ShinyGO 0.80 with FDR cutoff < 0.01.[2]

STRINGdb Search Parameters: Focusing on Tropomyosin (F4WSG5, inferred gene: G5I_08796) as the central node, we configured the network with the following parameters: network type set to full STRING network, activation of all interaction sources, a minimum required interaction score of medium confidence (0.400), and a maximum of 10 interactors in the first shell and 20 interactors in the second shell.

Method development of deciphering surface layer secretome. In our prior study, we successfully identified a protein-enriched insect epicuticle attached to the chitin structures of *A. echinatior* using scanning electron microscopy (SEM), suggesting a potential link between this protein layer and the biomineralization process. Unlike biomineralization in marine animals, which is associated with mineral precipitations from oceanic sources, terrestrial animals face challenges in absorbing metal ions from the atmosphere to form mineral layers. Initially, we hypothesized that the fungus gardens associated with *A. echinatior* might play an auxiliary role in this process. However, our current experiments have demonstrated that these fungus gardens do not contribute biologically to biomineralization. As a result, understanding the protein composition of this protein layer becomes essential for elucidating the biomineralization process in *A. echinatior*.

In previous attempts to isolate and analyze this protein layer, we employed both a mouthwash solution and high-concentration sodium hydroxide (NaOH) to dissolve and remove the proteins from the chitin structure. However, both methods presented challenges for proteomic analysis. The mouthwash, containing detergents, complicated protein extraction and purification, hindering subsequent mass spectrometry analysis. Sodium hydroxide, on the other hand, induced vigorous hydrolysis, leading to the identification of proteins only at the amino acid level.[3] Therefore, developing a milder yet effective method for profiling the protein composition is crucial.

To address this, we initially tested a diluted 0.1 N hydrochloric acid (HCl) method. Ants were placed in tubes containing 150 µL of HCl solution and agitated at 4°C for 0, 30, 60, and 90 min. After agitation, the ants were removed, and the solutions were neutralized with 8M urea with 50 mM Tris buffer to pH 8 for protein concentration measurement. Buffer-only controls served as negative controls for protein dissolution. However, nanodrop analysis of these samples revealed low and indistinguishable A280 values from the background, along with high A260/280 ratios, indicating poor protein extraction quality (**Supplemental Data S1, Extrac_first_time|major minor**).

To improve extraction efficiency, we extended the duration of HCl treatment and incorporated sonication during agitation. We tested control, 2h, 4h, 7h, and 10h agitation/sonication at 4°C. After protein dissolution, the solutions were neutralized and subjected to proteomic analysis. The remaining bodies of *A. echinatior* were fixed in methanol, and SEM images were captured to confirm protein removal. Visual inspection of SEM images demonstrated that the white protein layers gradually peeled off from the chitin skeleton over time (**Figure S5A**). By 7h of HCl treatment, the protein layers were almost completely removed, exposing the chitin skeleton.

We then analyzed the protein composition of the extraction solutions following buffer neutralization, trypsin digestion, and sample desalting. The Venn diagram in **Figure S5B** illustrates the number of protein identifications (IDs) in each solution. A clear increasing trend in IDs was observed from the 2nd to 7th hour (365, 1252, and 1373 proteins, respectively), followed by a decline at the 10th hour (959 proteins), consistent with our SEM observations. The drop in IDs at the 10th hour likely resulted from the extensive hydrolysis of dissolved proteins, a phenomenon previously observed with prolonged extraction times using strong acids and bases. Based on these findings, we concluded that the optimal time frame for profiling the outer layer proteins from the chitin skeleton lies between the 4th and 7th hour of HCl treatment.

Extended proteome-wide analysis

The early adult stage of the leaf-cutter ant represents a dynamic developmental period in which the ant continues to mature to meet the demands of its colony role. For example, further fortification of the cuticle takes place during the early adult ant stage through sclerotization, which involves the cross-linking of proteins and chitin.[4] In our results, we found F4WD95 cuticle protein and chitin-binding type-2 domain- and C-type lectin

domain-containing proteins enriched in the callow worker stage. These results point to the maturation of the chitin skeleton in early development in *A. echinatior*. Laminins such as laminin subunit alpha and laminin subunit beta-1, which are essential components of the extracellular matrix, were highly abundant during the earliest adult stages but decreased in abundance with age. These proteins are involved in facilitating cell adhesion, migration, and differentiation while contributing to tissue structural integrity and signal transduction, suggesting that major structural remodeling is ongoing shortly after eclosion.[5]

Unexpectedly, two proteins in the major royal jelly protein (MRJP) family were enriched in the callow worker stage, where we observed high abundance in callows and lower abundance in the young adult stage, suggesting a potential role of these proteins very early after eclosion. Proteins in the MRJP family have been extensively characterized in bees, where they play an important role in larval nutrition and contribute to sex- and caste-determination.[6, 7] However, knowledge of MRJP family related proteins in ants remains limited. Our data is intriguing, considering that the leaf-cutter ants display distinct worker caste polymorphism, a trait that is not exhibited by the majority of the fungus-farming ant taxa.[8] However, we did not find the MRJP-related proteins in the external secretome, indicating that the role of these proteins may be specifically internal.

Extended secretome analysis

Beyond the muscle-related myosin, actin, and tropomyosin proteins, other ion-binding proteins were also enriched in both the whole-body proteome and the secretome. For instance, four and a half LIM domains protein 2 and LIM domain-containing protein, which both have predicted metal-ion binding ability, were consistently detected in our whole-body proteome and secretome-enriched protein results. These may serve as candidate genes for future studies investigating how leaf-cutter ants are able to incorporate heavy metals in order to form biominerals and other heavy-element biomaterials such as Zn-enriched mandibles.

Our study also highlighted key proteins with predicted involvement in antibacterial activities, stress responses, and symbiotic interactions, which are interesting considering the harsh biotic and abiotic environment that the surface of the ant cuticle is exposed to. GO analysis identified clusters linked to “Response to stress” and “Responses to other organisms”, and these clusters featured proteins such as peroxiredoxins, transferrins, laminins, and flotillins. Peroxiredoxins, a ubiquitous family of antioxidant proteins, are known in other insects to protect from oxidative damage by detoxifying reactive species through a redox regulatory mechanism.[9] Evidence suggests peroxiredoxins could enhance oxidative stress resistance, such as the contribution of *Drosophila* PRDX5 in extending lifespan, and may also support immune responses, as seen with PRDX5 upregulation in silkworms under stress.[10] Similarly, flotillins, highly conserved membrane-associated proteins, are implicated in lipid raft organization, endocytic trafficking, and signal transduction pathways, often linked to cellular stress responses. These predicted functions highlight their potential importance in insect physiology for reacting to harsh external environments. Additional enriched proteins included catalase and lethal essential for life (LEL) proteins are critical for cellular survival and function. Catalase acts as a key antioxidant enzyme, decomposing hydrogen peroxide into water and oxygen, thereby protecting cells from oxidative damage and maintaining redox homeostasis. LEL proteins, essential for viability, participate in fundamental processes such as transcription, translation, cell division, and metabolic regulation.

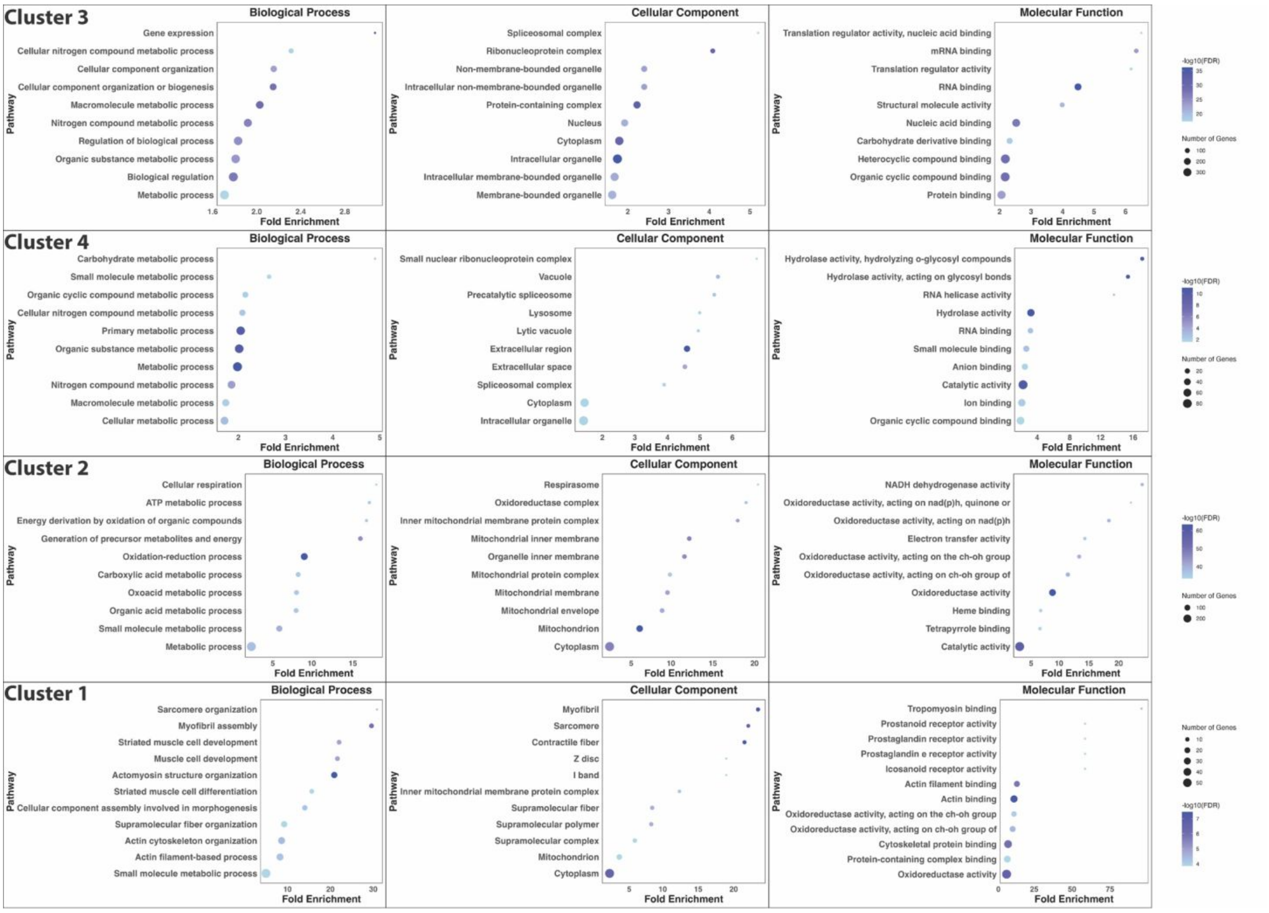

Figure S1. GO Functional enrichment analysis for each cluster of proteome-wide analysis, showing significant biological processes, cellular components, and molecular functions associated with the proteins within each cluster. Extended GO enrichment analysis from **Figure 1**.

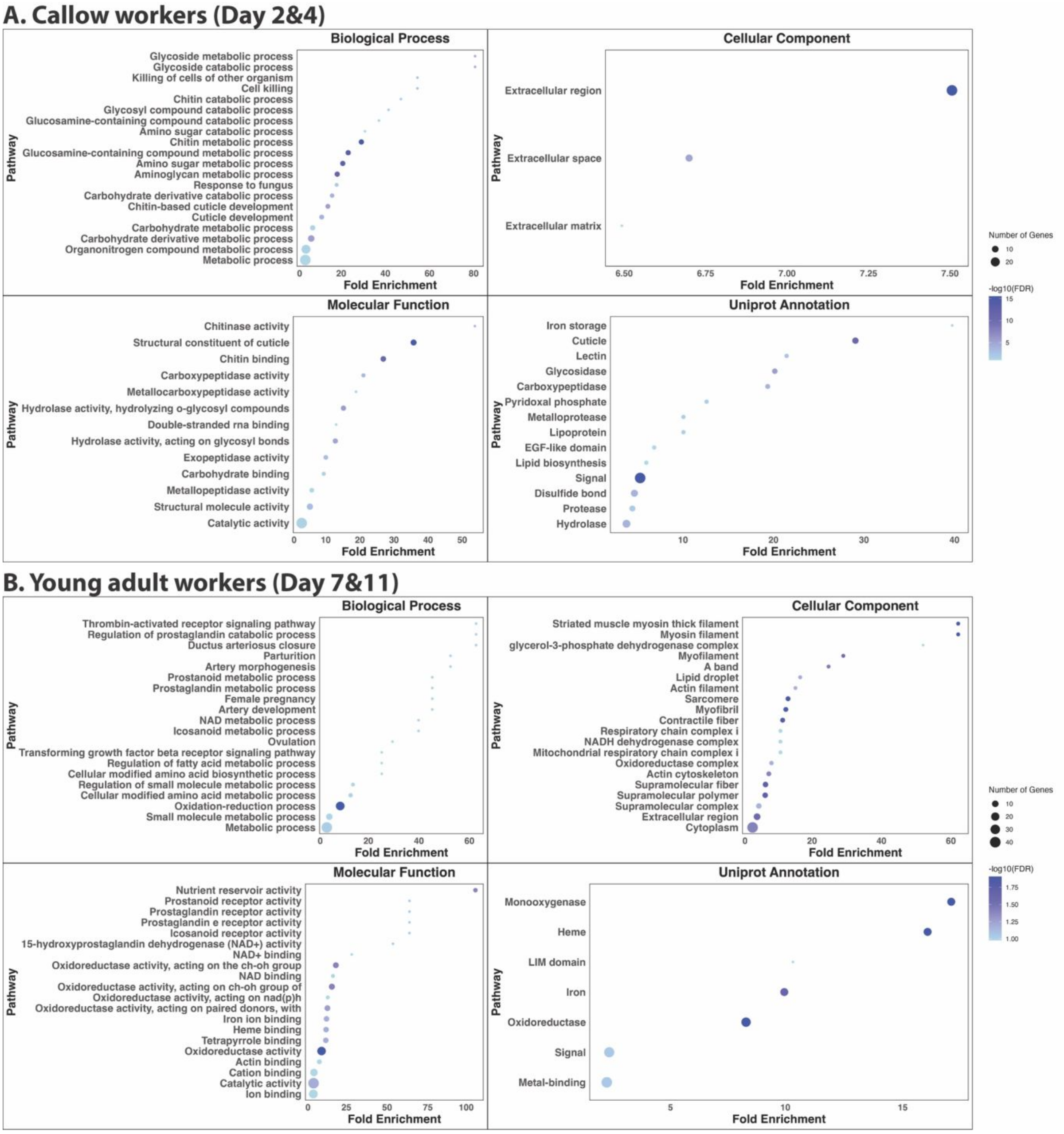

Figure S2. GO Analysis of the proteomes of callow (Day 2 and 4) vs. young adult (Day 7 and 11) *A. echinatior* workers. Extended GO enrichment analysis for the volcano plot from **Figure 2**. The completed GO BP, CC, MF, and Uniprot annotation for proteins increased accumulation in Callow workers and Young adult workers, respectively.

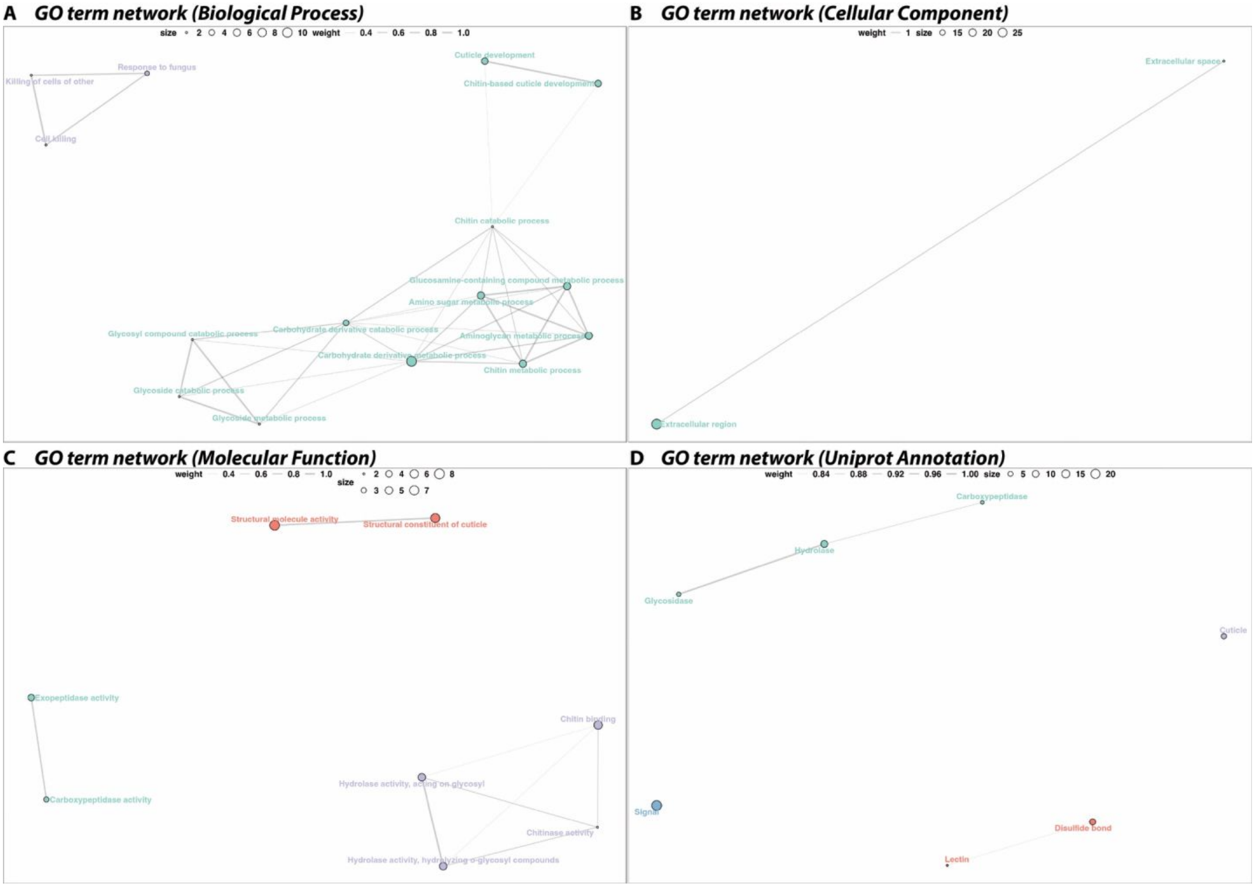

Figure S3. GO term network of the proteins exhibiting higher detected abundance in the "Callow". (A-D) Biological process, cellular component, molecular function, and Uniport annotation were visualized respectively. The original results can be found in supplementary data 2.

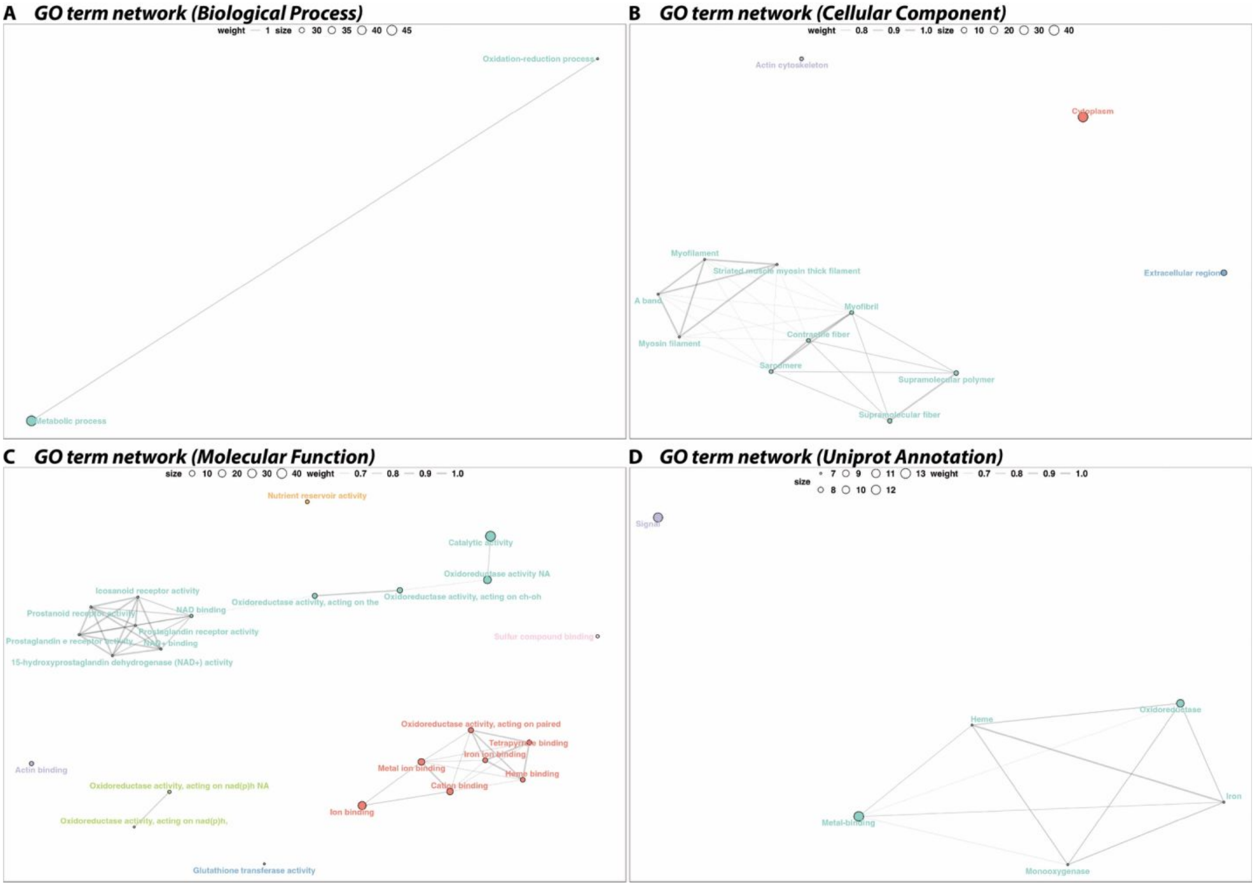

Figure S4. GO term network of the proteins exhibiting higher detected abundance in the “Young Adult”. (A-D) Biological process, cellular component, molecular function, and Uniport annotation were visualized respectively. The original results can be found in supplementary data 2.

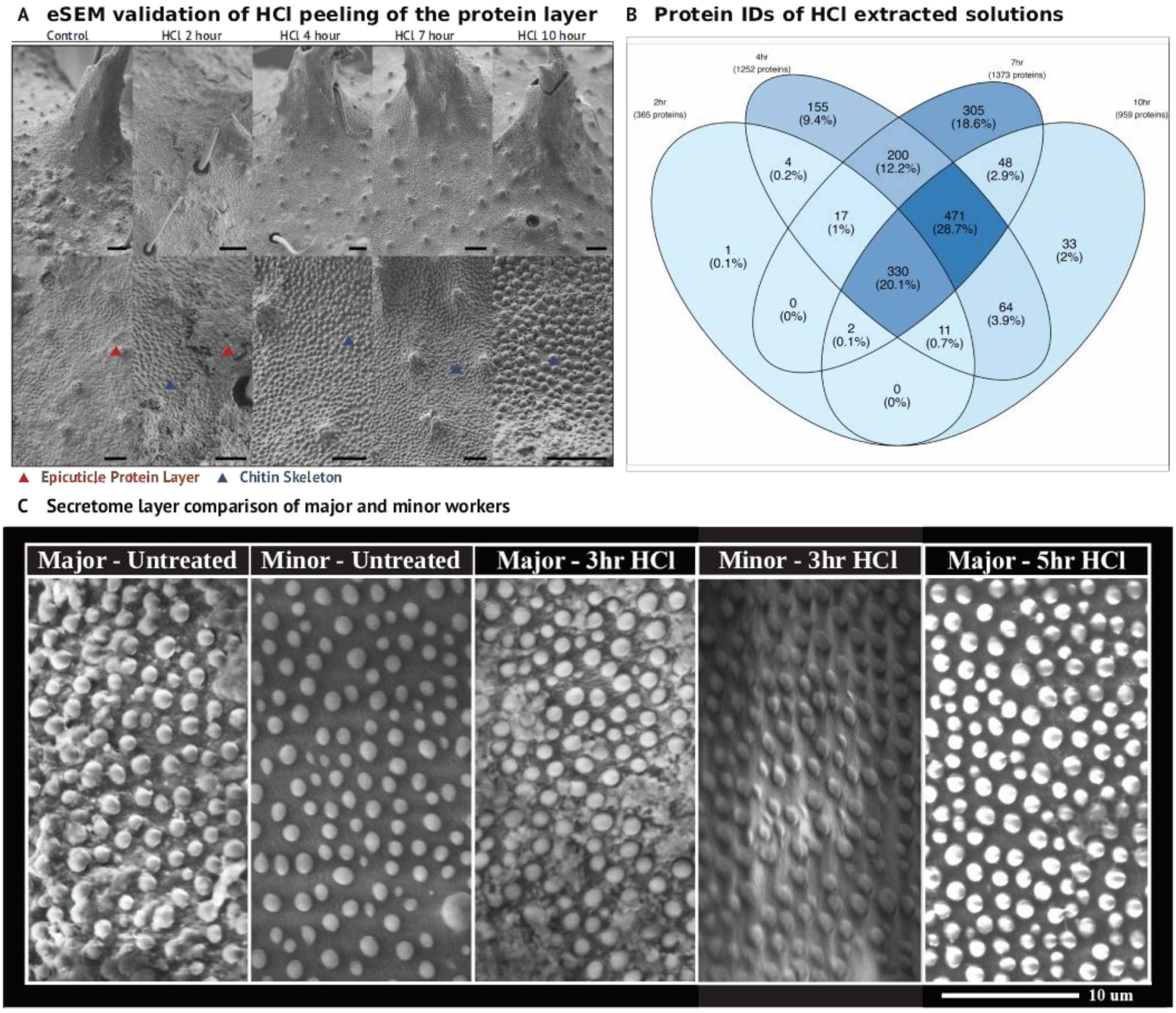

Figure S5. SEM Validation and Protein Extraction Over Time (A) SEM Validation of HCl Peeling(Top – zoom-out, scale bar, 20 µm. Bottom – zoom-in, scale bar, 10 µm.): The scanning microscopy (SEM) images validate the progressive peeling of the epicuticle protein layer from the chitin skeleton over different HCl treatment times (2hto 10h). Red arrowheads indicate the epicuticle protein layer, while blue arrowheads denote the chitin skeleton. (B) Venn Diagram of Protein IDs from HCl Extracted Solutions: The Venn diagram illustrates the overlap of protein identifications from HCl-extracted solutions treated for 2h, 4h, 7h, and 10h. A total of 330 proteins were consistently identified across all time points, indicating robust extraction and consistent protein recovery. (C) SEM images of cuticular surface of major and minor *A. echinatior* worker ants with or without HCl treatment.

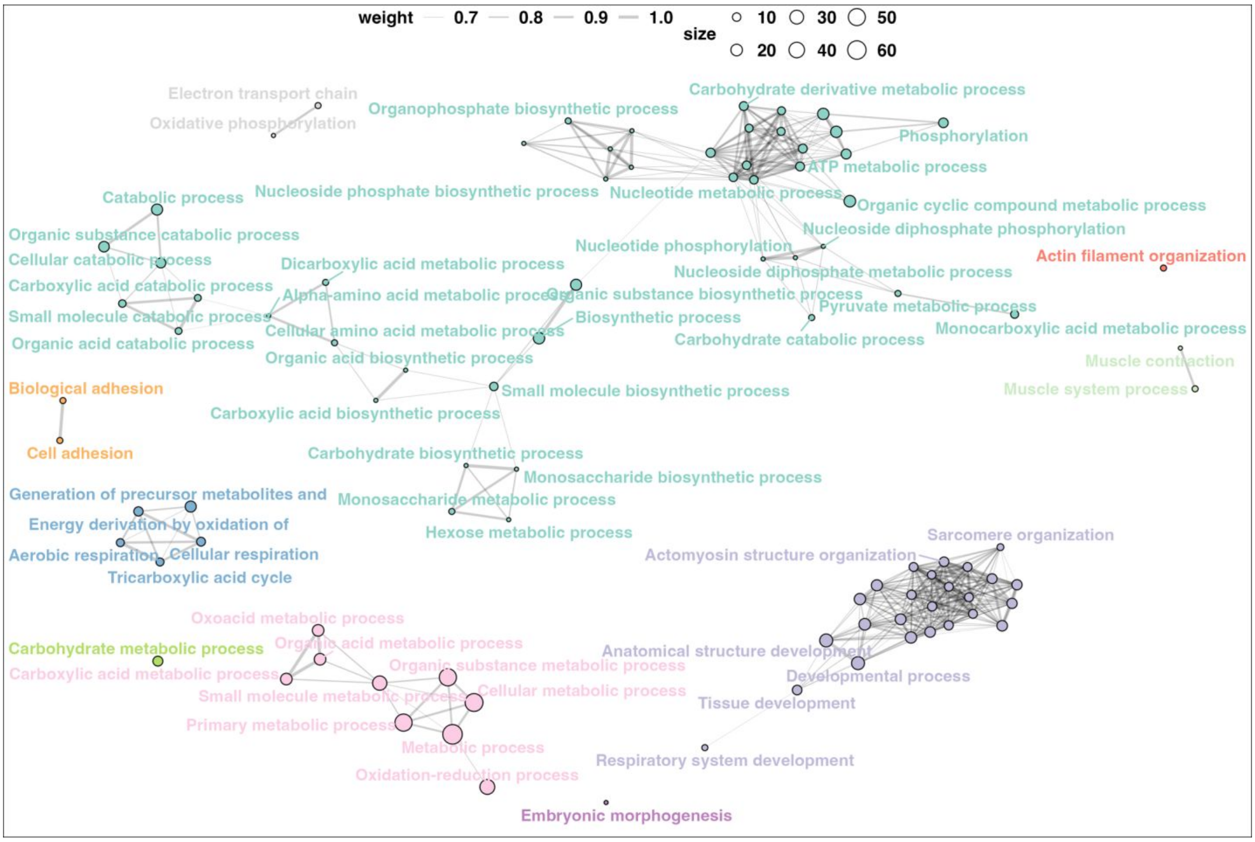

Figure S6. GO term network BP(5h, p < 0.001)

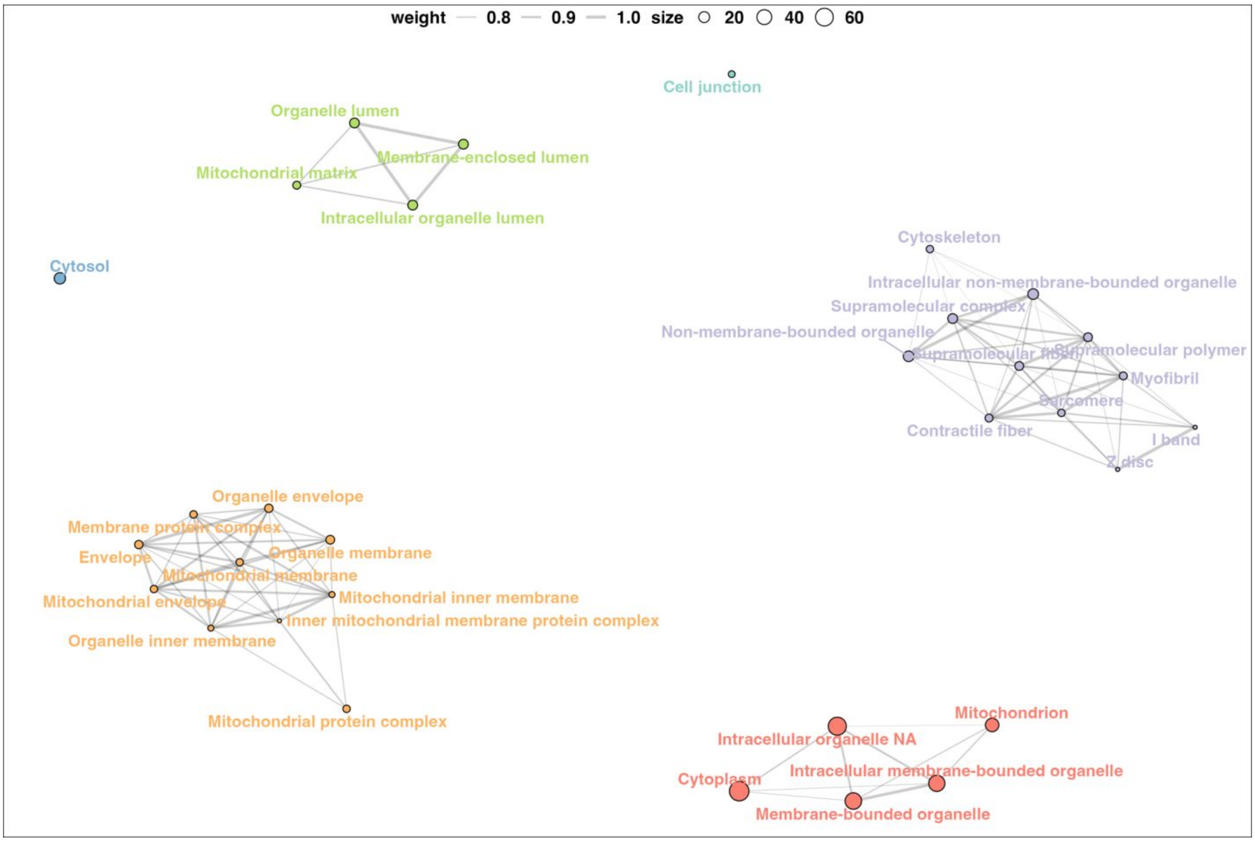

Figure S7. GO term network CC(5h, p < 0.001)

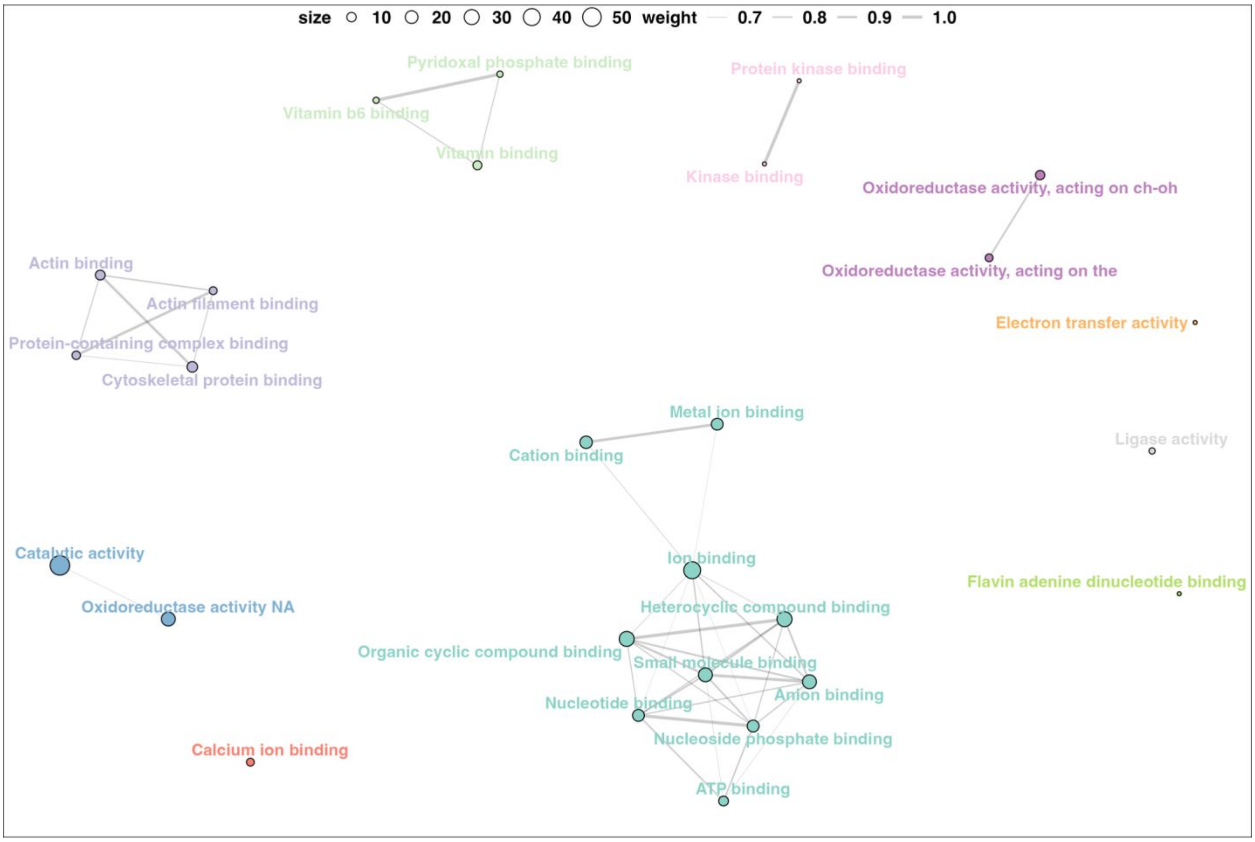

Figure S8. GO term network MF(5h, $p < 0.001$)

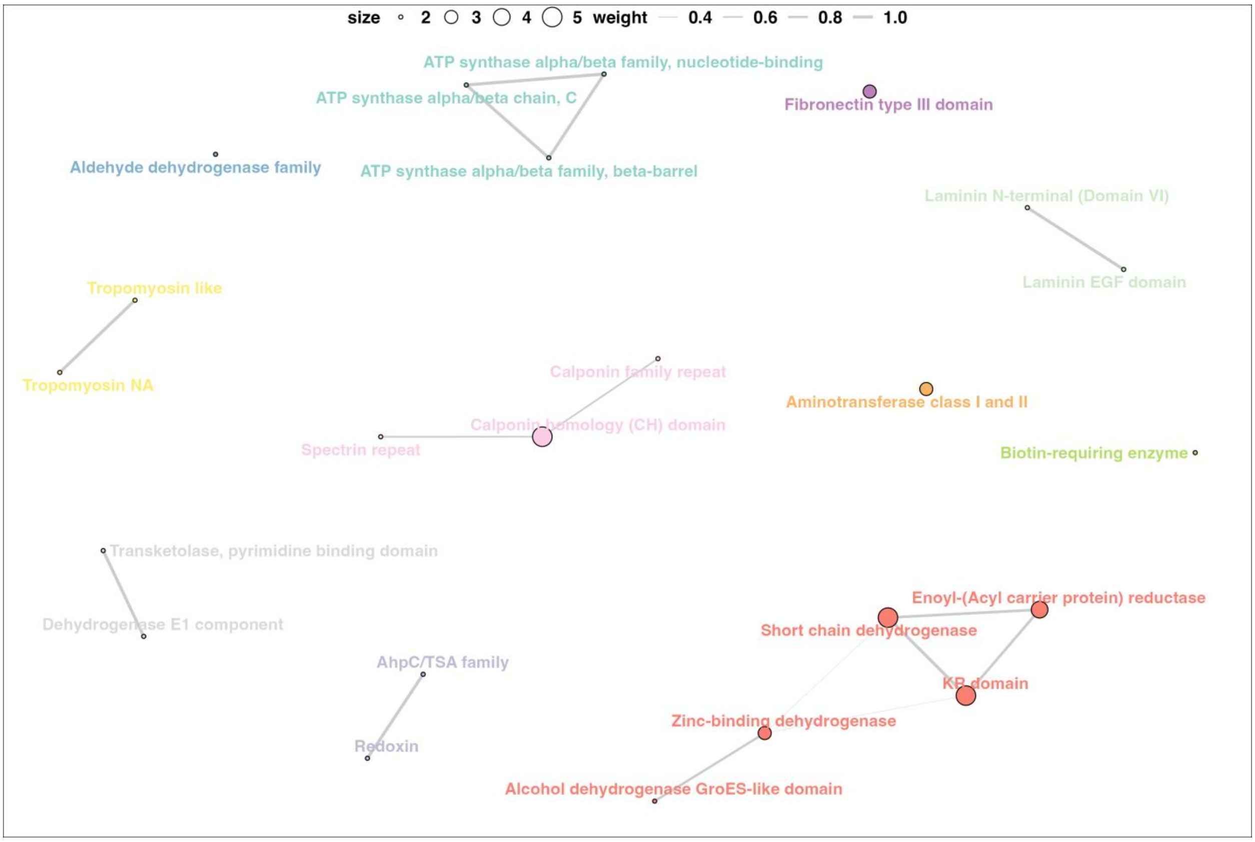

Figure S9. GO term network Pfam(5h, $p < 0.001$)

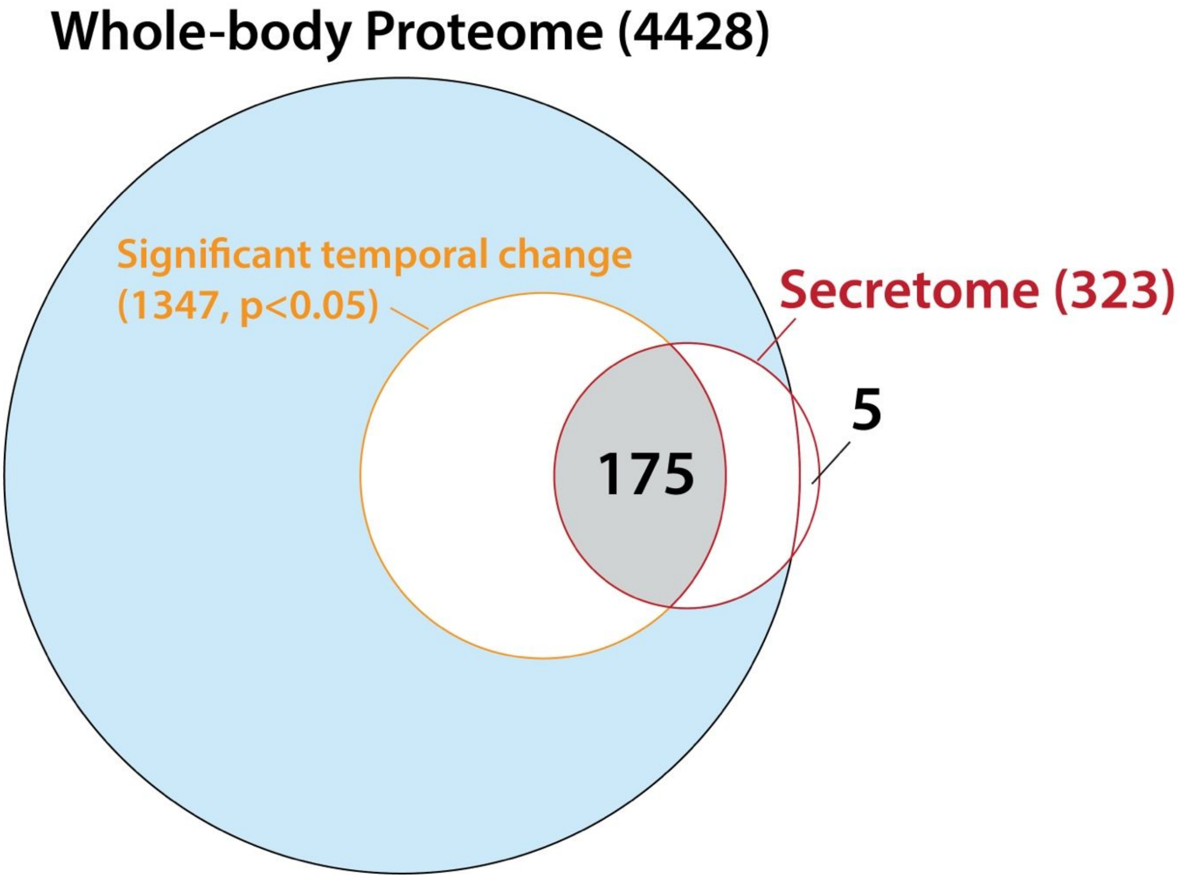

Figure S10. Venn diagram of the secretome protein IDs in comparison to the whole-body proteome IDs. In total, the whole-body proteome data resulted in 4428 proteins with 1347 significantly altered at different ages ($p < 0.05$), where the secretome data resulted in 323 proteins with the 175 proteins intersected showing significant temporal changes.

G5I_07096	F4WMW0_ACREC	Peroxiredoxin-6	Thiol-specific peroxidase that catalyzes the reduction of hydrogen peroxide and organic hydroperoxides to water and alcohols, respectively.	#Antioxidant #Oxidoreductase	https://www.uniprot.org/uniprotkb/F4WMW0/entry
G5I_10028	F4WVS6_ACREC	thioredoxin-dependent peroxiredoxin	Thiol-specific peroxidase that catalyzes the reduction of hydrogen peroxide and organic hydroperoxides to water and alcohols, respectively.	#Antioxidant #Oxidoreductase	https://www.uniprot.org/uniprotkb/F4WVS6/entry
G5I_12297	F4X1X8_ACREC	Peroxiredoxin-5	Thiol-specific peroxidase that catalyzes the reduction of hydrogen peroxide and organic hydroperoxides to water and alcohols, respectively. Plays a role in cell protection against oxidative stress by detoxifying peroxides and as sensor of hydrogen peroxide-mediated signaling events.	#Antioxidan #Oxidoreductase #Peroxidase	https://www.uniprot.org/uniprotkb/F4X1X8/entry

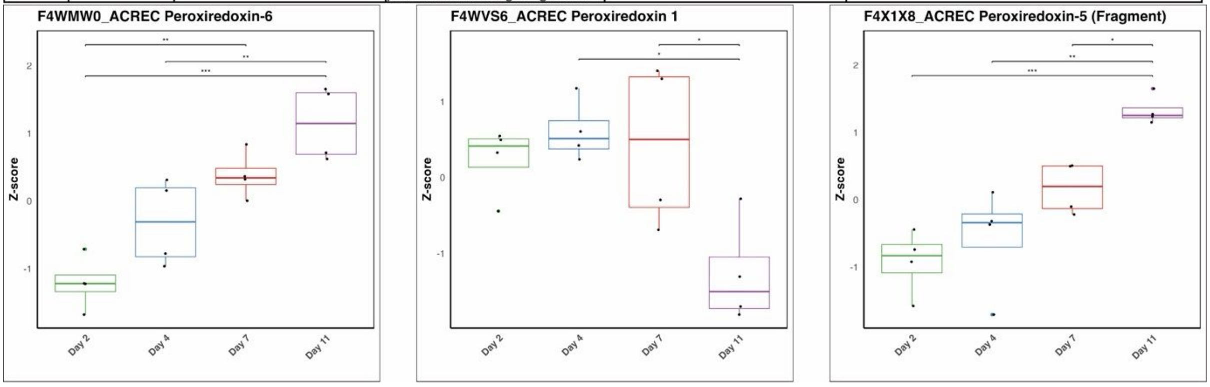

Figure S11. Proteins categorized under the pink cluster with the biological functions of responding to external environmental stimulus. Three peroxiredoxin proteins with the antioxidant activities were identified, and their quantitative alterations across different developmental ages were plotted.

G5I_02004	F4W957_ACREC	Transferrin	Transferrins are iron binding transport proteins which bind Fe^{3+} ion in association with the binding of an anion, usually bicarbonate.	#Ion transport #Iron transport #Metal-binding	https://www.uniprot.org/uniprotkb/F4W957/entry

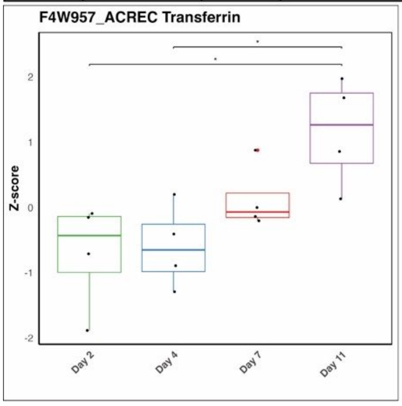

Figure S12. Proteins categorized under the pink cluster with the biological functions of responding to external environmental stimulus. The transferrin protein with the ion transport and metal-binding activities was identified, and its quantitative alterations across different developmental ages were plotted.

tr_F4W957_F4W957_ACREC
Predicted localizations: Extracellular
Predicted membrane association: Soluble
Predicted signals: Signal peptide

Localization	Cytoplasm	Nucleus	Extracellular	Cell membrane	Mitochondrion	Plastid	Endoplasmic reticulum	Lysosome/Vacuole	Golgi apparatus	Peroxisome
Probability	0.2018	0.1157	0.7063	0.2046	0.0613	0.0078	0.1670	0.3015	0.0925	0.0005

Membrane association	Peripheral	Transmembrane	Lipid anchor	Soluble
Probability	0.4830	0.1240	0.2640	0.7240

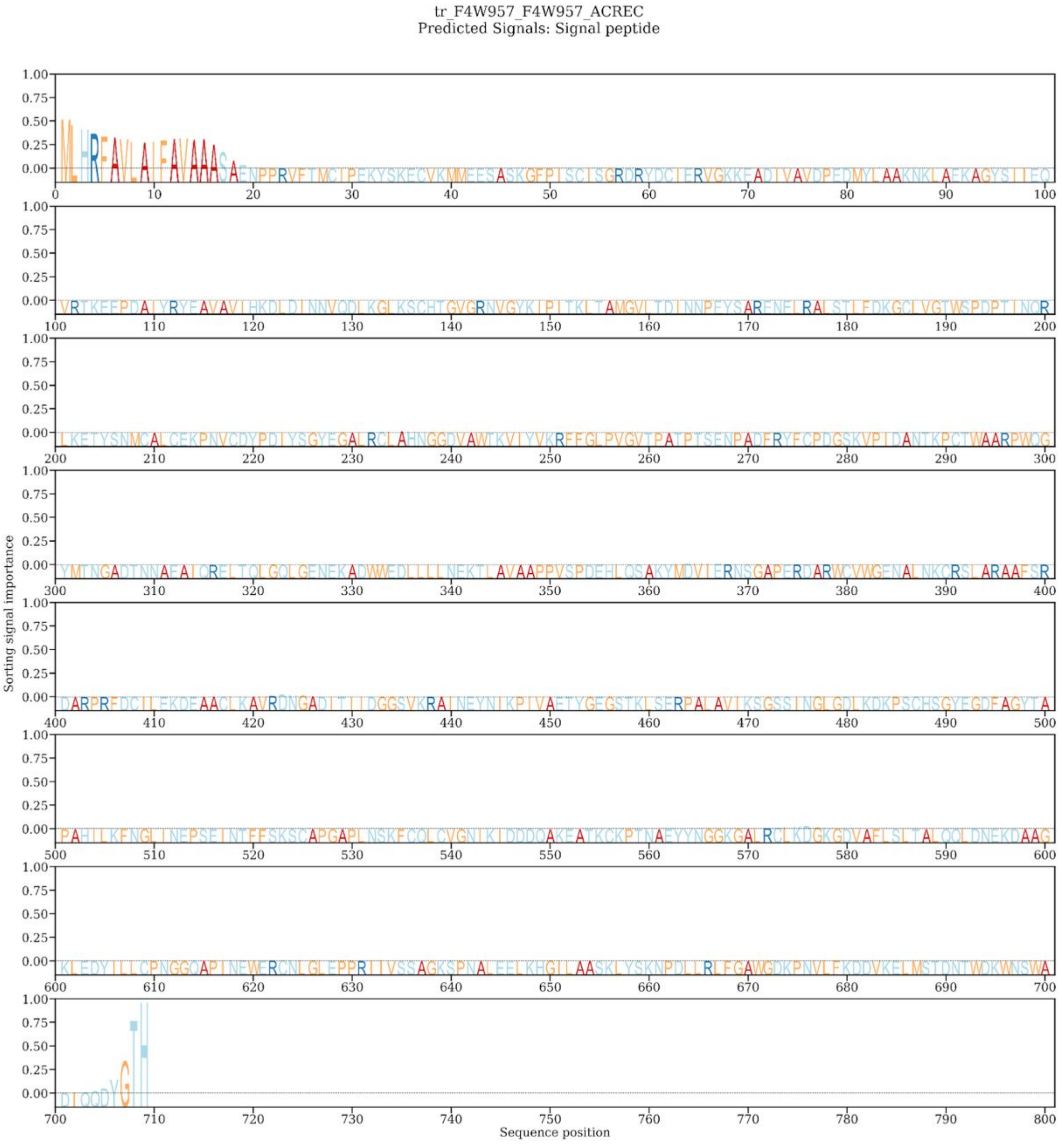

Figure S13. F4W957 Transferrin as an example of bioinformatic tool DeepLoc 2.1-predicted secretome protein with the signal peptide sequence and predicted to be in the extracellular region.

G5I_10924	F4WY67_ACREC	Laminin subunit alpha	#Basement membrane #Extracellular matrix #Secreted	https://www.uniprot.org/uniprotkb/F4WY67/entry
G5I_12012	F4X156_ACREC	Neuroglian		https://www.uniprot.org/uniprotkb/F4X156/entry
G5I_12920	F4X3L2_ACREC	Laminin subunit beta-1	tissue development animal organ morphogenesis	https://www.uniprot.org/uniprotkb/F4X3L2/entry
G5I_08371	F4WRC5_ACREC	Flotillin-2	#Cell adhesion	https://www.uniprot.org/uniprotkb/F4WRC5/entry

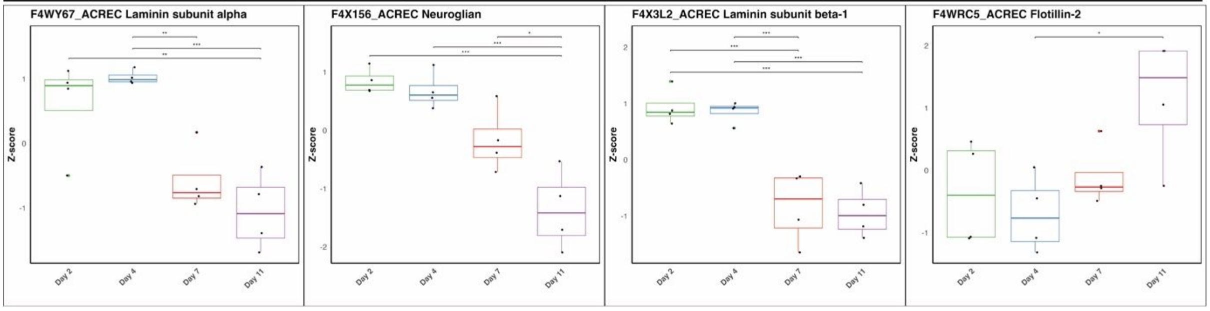

Figure S14. Proteins categorized under the pink cluster with the biological functions of developmental adaptions. Tissue developmental and cell adhesion proteins were identified, and their quantitative alterations across different developmental ages were plotted.

tr_F4WY67_F4WY67_ACREC
Predicted localizations: Extracellular, Cell membrane
Predicted membrane association: Soluble
Predicted signals: Signal peptide

Localization	Cytoplasm	Nucleus	Extracellular	Cell membrane	Mitochondrion	Plastid	Endoplasmic reticulum	Lysosome/Vacuole	Golgi apparatus	Peroxisome
Probability	0.2759	0.1113	0.7715	0.5819	0.1021	0.0333	0.1461	0.1952	0.1663	0.0059

Membrane association	Peripheral	Transmembrane	Lipid anchor	Soluble
Probability	0.5850	0.2110	0.0720	0.7040

Figure S15. F4WY67 Laminin subunit alpha as an example of bioinformatic tool DeepLoc 2.1-predicted secretome protein with the signal peptide sequence and predicted to be in the extracellular region.

G5I_01100	F4W6K5_ACREC	Catalase	Catalyzes the degradation of hydrogen peroxide (H2O2) generated by peroxisomal oxidases to water and oxygen, thereby protecting cells from the toxic effects of hydrogen peroxide.	#Oxidoreductase #Peroxidase	https://www.uniprot.org/uniprotkb/F4W6K5/entry
G5I_08477	F4WRM4_ACREC	Protein lethal(2)essential for life		#Stress response	https://www.uniprot.org/uniprotkb/F4WRM4/entry

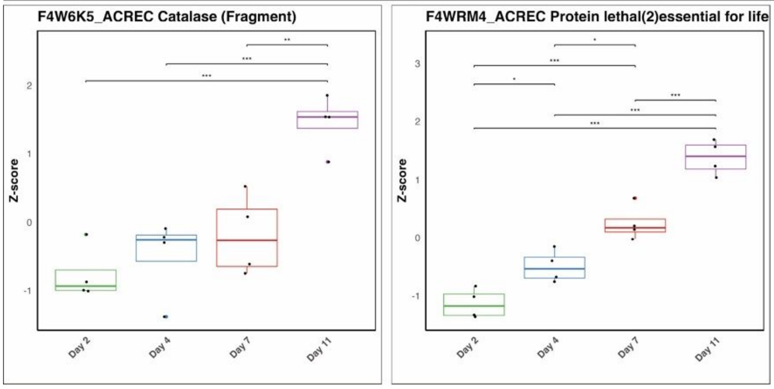

Figure S16. Proteins categorized under the purple cluster ("Response to stress") with the biological functions of stress response. Catalase and protein lethal essential for life proteins were identified, and their quantitative alterations across different developmental ages were plotted.

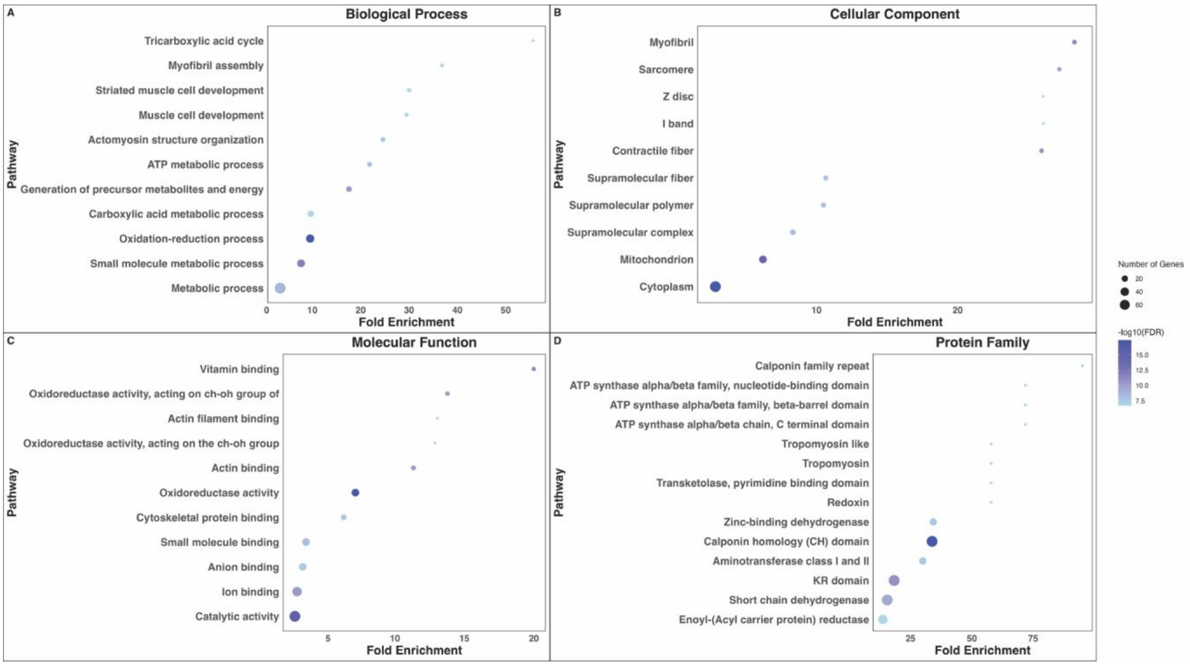

Figure S17. GO Functional enrichment analysis for secretome characterization, extended data from **Figure 3B**.

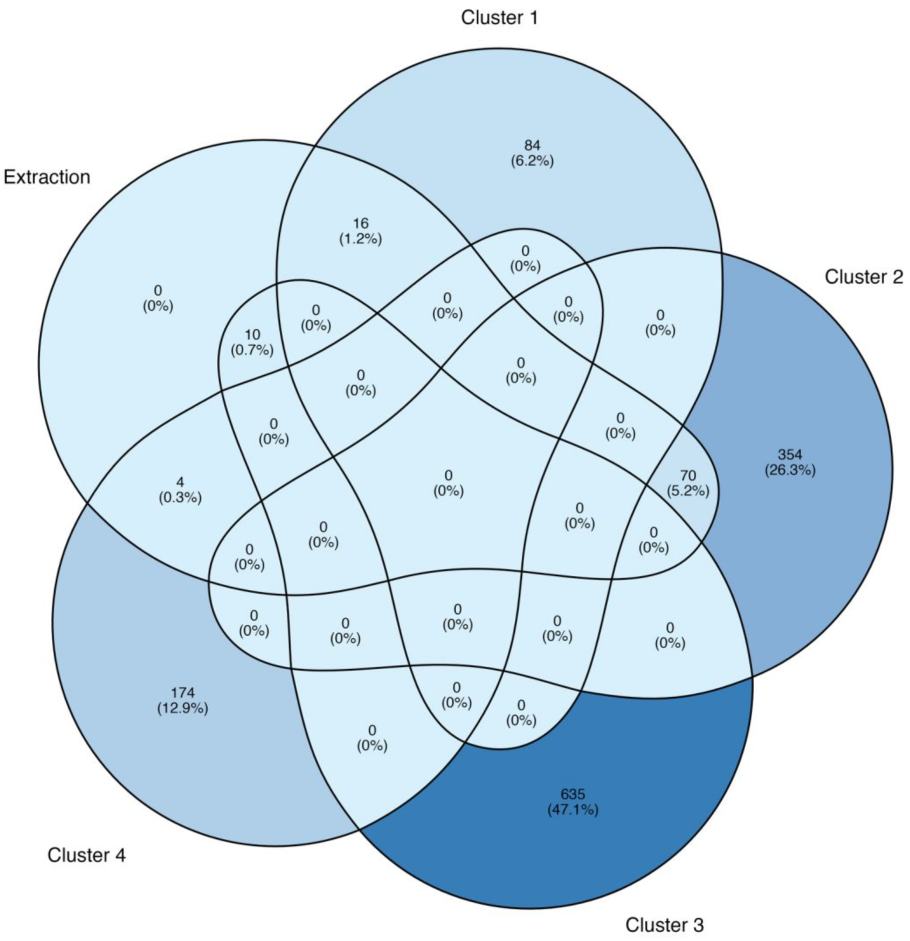

Figure S18. The Venn diagram shows the intersection of proteins extracted by HCl treatment (5h extraction w/ 100 proteins showing significant alterations in prior whole-body proteome experiments, labeled as "Extraction") with proteins found in each cluster from the global proteome analysis (Clusters 1 to 4). Cluster 2 (70, 5.2%) and Cluster 1 (16, 1.2%) contain the highest overlap with the extracted proteins, indicating that proteins in these clusters are significantly represented in the acid-extracted fraction.

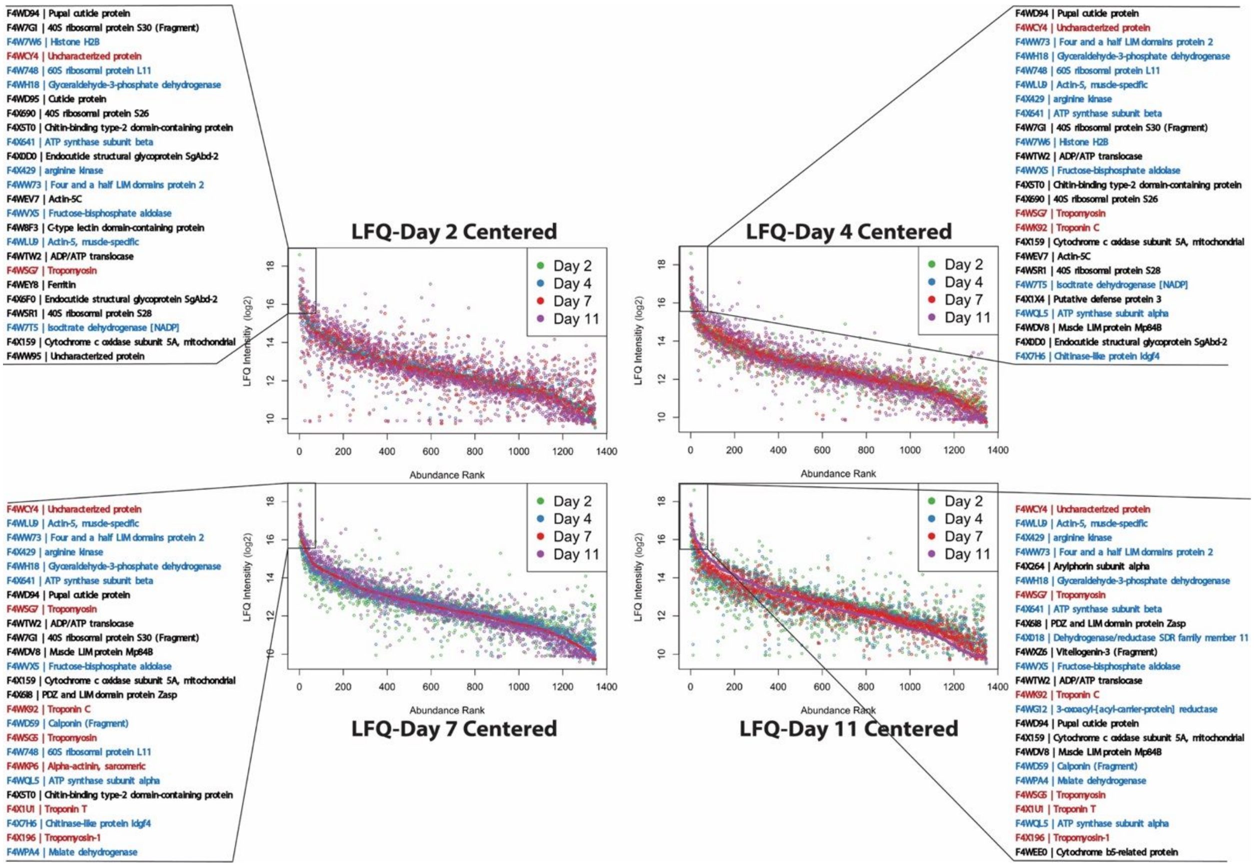

Figure S19. LFQ intensity-based comparison of protein abundance across developmental ages. (A-D) Centered LFQ intensity plots for Day 2, 4, 7, and 11, highlighting specific proteins with significant changes in abundance. Proteins involved in the identified tropomyosin family are emphasized and highlighted in red. The other identified external secretome proteins are highlighted in blue.

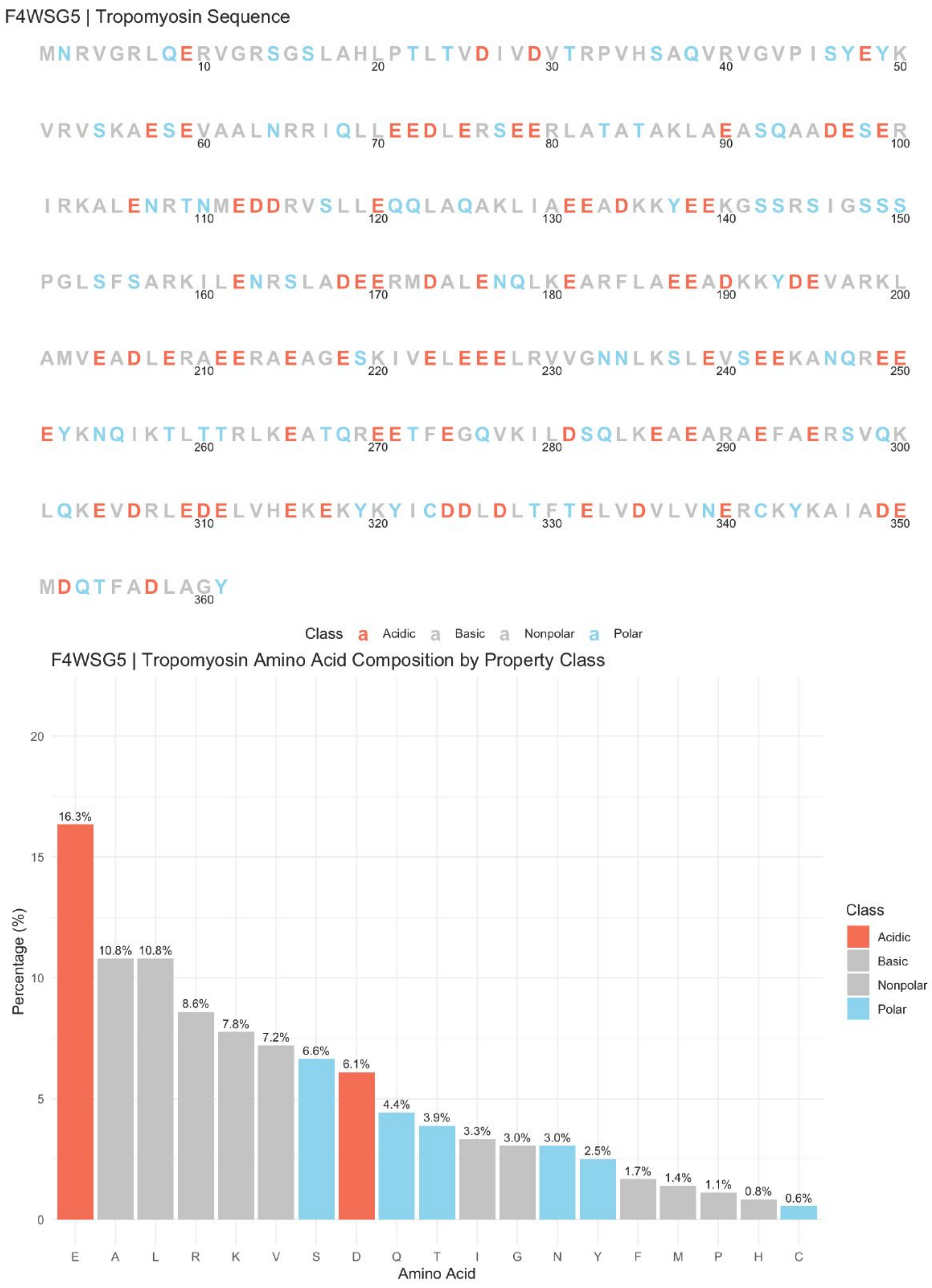

Figure S20. F4WSG5 Tropomyosin protein sequence color-coded by property classes. Acidic and polar amino acid residues are colored with red and blue, respectively.

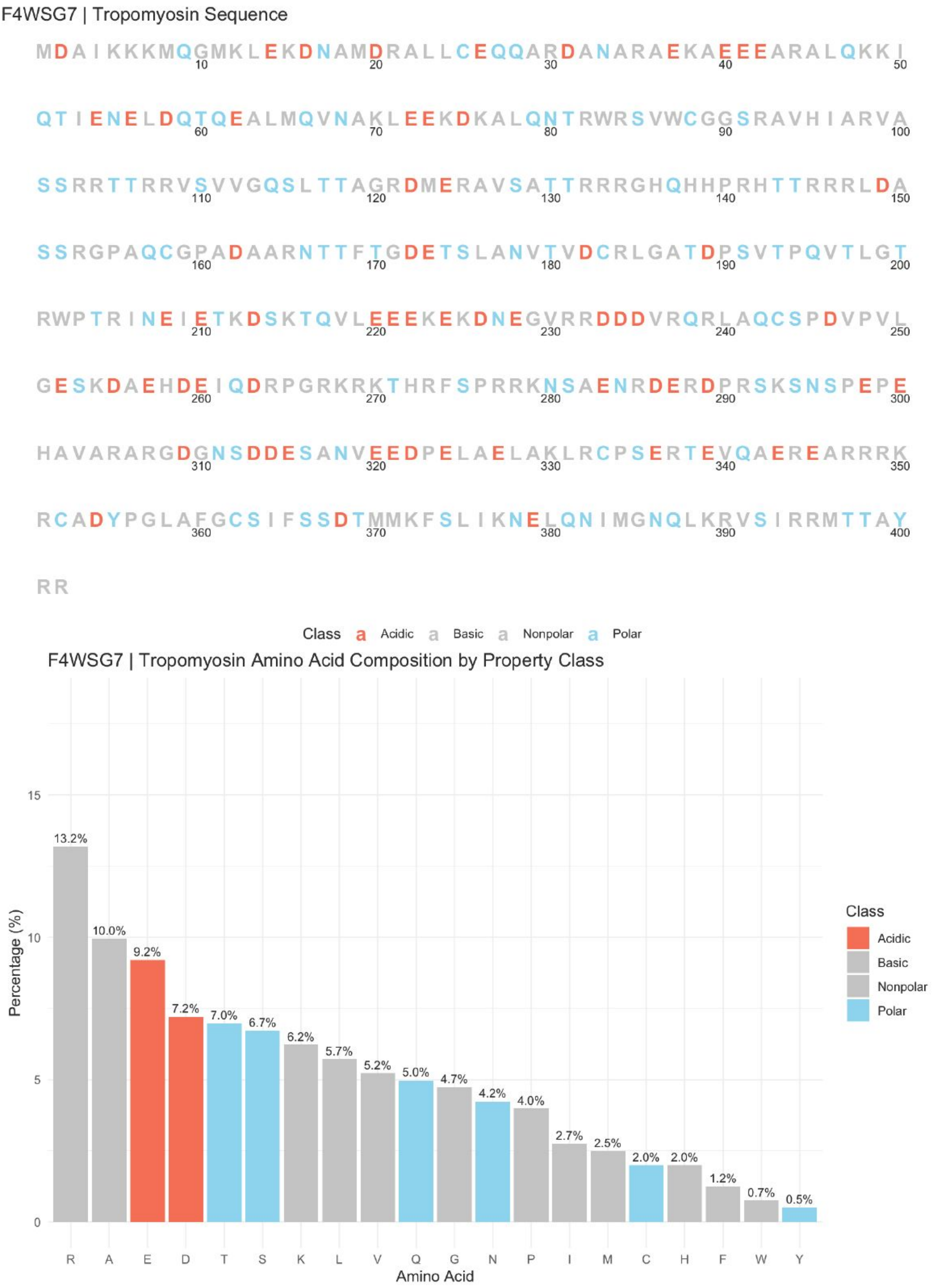

Figure S21. F4WSG7 Tropomyosin protein sequence color-coded by property classes. Acidic and polar amino acid residues are colored with red and blue, respectively.

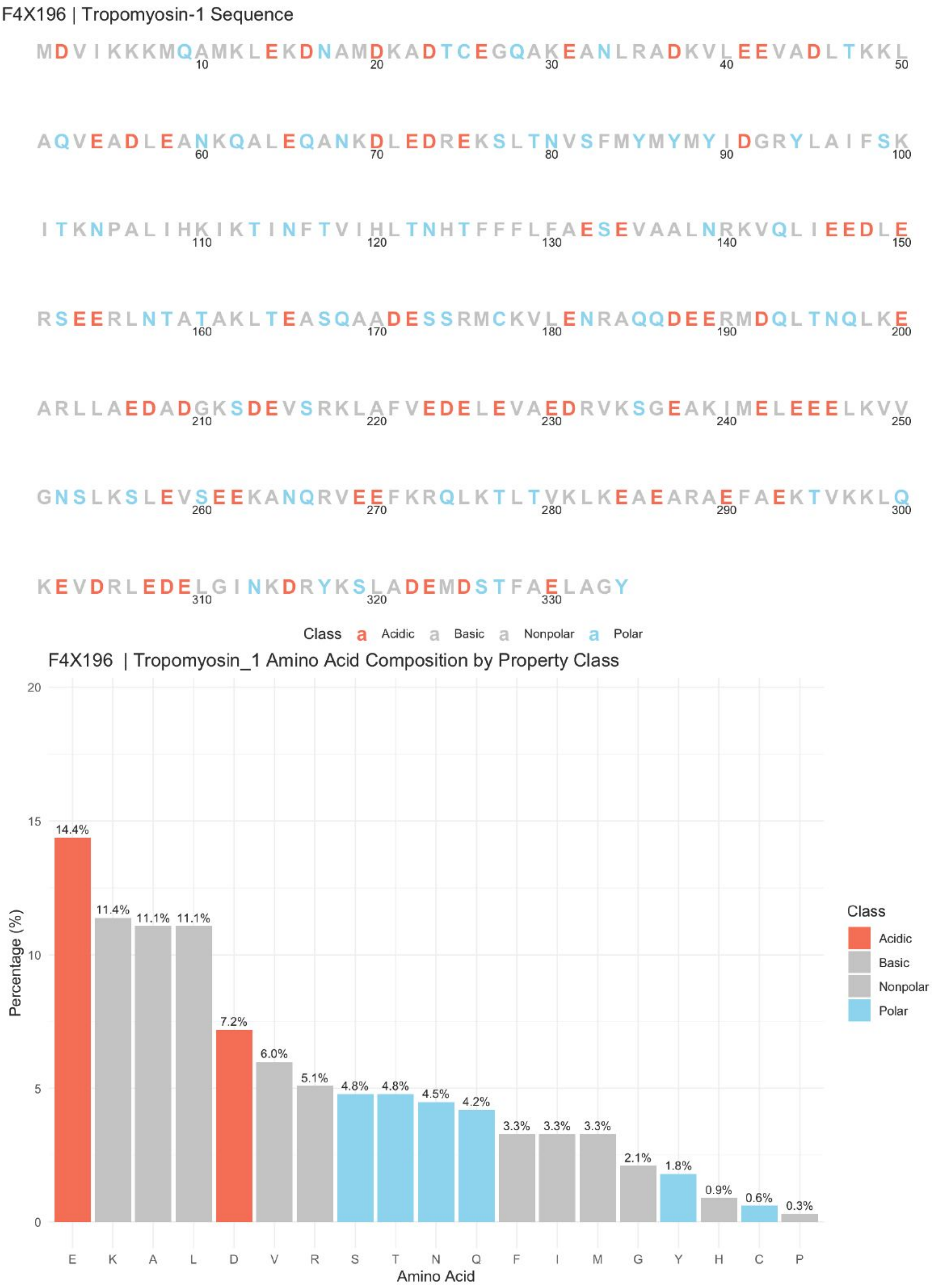

Figure S22. F4X196 Tropomyosin-1 protein sequence color-coded by property classes. Acidic and polar amino acid residues are colored with red and blue, respectively.

Supplementary Reference

(1) Gerault, M. A.; Camoin, L.; Granjeaud, S. DIAgui: a Shiny application to process the output from DIA-NN. *Bioinform Adv* **2024**, *4* (1), vbae001. DOI: 10.1093/bioadv/vbae001

(2) Ge, S. X.; Jung, D.; Yao, R. ShinyGO: a graphical gene-set enrichment tool for animals and plants. *Bioinformatics* **2019**, *36* (8), 2628--2629. DOI: 10.1093/bioinformatics/btz931 , pmid = 31882993 , pmcid = PMC7178415

(3) Li, H.; Sun, C. Y.; Fang, Y.; Carlson, C. M.; Xu, H.; Ješovnik, A.; Sosa-Calvo, J.; Zarnowski, R.; Bechtel, H. A.; Fournelle, J. H.; et al. Biomineral armor in leaf-cutter ants. *Nature Communications 2020 11:1* **2020**, *11*, 1--11. DOI: 10.1038/s41467-020-19566-3 , pmid = 33235196

(4) Andersen, S. O. Insect cuticular sclerotization: a review. *Insect Biochem Mol Biol* **2010**, *40* (3), 166-178. DOI: 10.1016/j.ibmb.2009.10.007

(5) Aumailley, M. The laminin family. *Cell Adh Migr* **2013**, *7* (1), 48-55. DOI: 10.4161/cam.22826

(6) Santos, K. S.; dos Santos, L. D.; Mendes, M. A.; de Souza, B. M.; Malaspina, O.; Palma, M. S. Profiling the proteome complement of the secretion from hypopharyngeal gland of Africanized nurse-honeybees (Apis mellifera L.). *Insect Biochem Mol Biol* **2005**, *35* (1), 85-91. DOI: 10.1016/j.ibmb.2004.10.003

(7) Schmitzova, J.; Klaudiny, J.; Albert, S.; Schroder, W.; Schreckengost, W.; Hanes, J.; Judova, J.; Simuth, J. A family of major royal jelly proteins of the honeybee Apis mellifera L. *Cell Mol Life Sci* **1998**, *54* (9), 1020-1030. DOI: 10.1007/s000180050229

(8) Mehdiabadi, N. J.; Schultz, T. R. Natural history and phylogeny of the fungus-farming ants (Hymenoptera: Formicidae: Myrmicinae: Attini). *Myrmecol News* **2010**, *13*, 37-55.

(9) Knoops, B.; Argyropoulou, V.; Becker, S.; Ferte, L.; Kuznetsova, O. Multiple Roles of Peroxiredoxins in Inflammation. *Mol Cells* **2016**, *39* (1), 60-64. DOI: 10.14348/molcells.2016.2341

(10) Shi, G. Q.; Yu, Q. Y.; Shi, L.; Zhang, Z. Molecular cloning and characterization of peroxiredoxin 4 involved in protection against oxidative stress in the silkworm Bombyx mori. *Insect Mol Biol* **2012**, *21* (6), 581-592. DOI: 10.1111/j.1365-2583.2012.01161.x
